# Mechanism study of low-ambient-pressure effects on the efficiency of cavitation erosion mitigation by air bubbles in gas-liquid two-phase flow

**DOI:** 10.1016/j.ultsonch.2026.108045

**Published:** 2026-09-08

**Authors:** Tong Qu, Jing Luo, Jie Li, Guihua Fu, Huamei Yang, Weilin Xu

**Affiliations:** a State Key Laboratory of Hydraulics and Mountain River Engineering, Sichuan University, Chengdu 610065, China; b Hubei Provincial Key Laboratory of Hydraulic Materials and Applied Technology, Three Gorges Corporation, Wuhan 430010, China

**Keywords:** Cavitation bubble, Air bubble, Low ambient pressure, Microjet, Shockwaves, Aeration-based cavitation erosion mitigation

## Abstract

Ambient pressure variations alter cavitation bubble dynamics, thereby affecting cavitation erosion on solid boundaries and the efficiency of aeration-based mitigation. In this study, the cavitation erosion mitigation efficiency and underlying mechanisms of entrained air bubbles in gas–liquid two-phase flow under varying ambient pressures were systematically investigated by combining macroscopic investigation and mesoscopic analysis. Ultrasonic cavitation experiments were conducted at ambient pressures of 55–95 kPa to quantify the erosion mitigation characteristics of aeration. The results showed that, under aerated conditions, the eroded-area ratio, pit depth, mean pit volume, cumulative mass loss and the erosion rate all decrease monotonically with decreasing ambient pressure, while the mitigation efficiency of aeration increases correspondingly. To elucidate the underlying mechanism, corona discharge system was employed to generate cavitation bubbles, enabling direct observation of the air bubbles interaction with cavitation bubbles. It was found that air bubbles significantly reduced the maximum velocity of microjet and attenuating the intensity of shockwaves impinging on the wall. Furthermore, both the maximum microjet velocity and the shockwave intensity decreased with decreasing ambient pressure under aerated conditions. The macroscopic trends in cavitation erosion mitigation of aeration and the mesoscopic effects of air bubbles on cavitation bubble dynamics together reveal the underlying mitigation mechanisms. They explain how aeration reduces cavitation erosion on solid boundaries exposed to high-speed water flow under high-altitude, low atmospheric pressure conditions. These findings offer theoretical support for applying aeration-based cavitation erosion mitigation in such conditions.

## 1 Introduction

Aeration has been widely recognized as an important means of mitigating cavitation erosion, with extensive applications in hydraulic engineering [1], [2], chemical engineering [3], [4], and ocean engineering [5], [6]. In hydraulic and ocean engineering, injecting air into cavitation regions effectively reduces cavitation erosion damage on hydraulic structures and propeller surfaces [7], [8]. In chemical engineering, aeration is commonly used to regulate cavitation intensity in sonochemical reactors [9], while also supporting industrial processes such as oxidative degradation [10] and wastewater treatment [11], [12]. Cavitation often occurs under different ambient pressure conditions. For example, low ambient pressure is common in high-speed water-release structures at high altitudes [13], [14]. It can also occur in sonochemical reactors operated under reduced pressure [15]. Such pressure variations alter cavitation bubble behavior [16] and its interactions with entrained air bubbles, thereby markedly influencing the cavitation erosion mitigation efficiency of aeration.

Since the application of aeration-based cavitation erosion mitigation techniques, researchers have extensively investigated the relationship between aeration characteristics and cavitation erosion mitigation efficiency at the macroscopic scale under atmospheric pressure. Early studies identified aeration concentration as a key parameter for evaluating cavitation erosion mitigation performance. Peterka [1] demonstrated that increasing air content significantly reduced cavitation erosion, highlighting the protective effect of aeration. Russell and Sheehan [17] further confirmed the pronounced suppressive effect of aeration on cavitation erosion, reporting that under certain conditions, aeration could substantially reduce cavitation erosion. Accordingly, critical aeration concentration became an important reference for subsequent research and engineering design. Further investigations indicated that mitigation efficiency is controlled by multiple aeration characteristics, including air concentration, bubble number, and spatial distribution. Wu and Luo [18] compared different air injection methods and found that, under an equivalent air supply area, multi-point injection provided stronger cavitation erosion mitigation than single-point injection. Recent studies have further identified air content and bubble size as key factors in cavitation erosion mitigation. Wu et al. [19] showed that higher aeration concentrations significantly enhanced mitigation efficiency and that smaller bubbles generally provided stronger mitigation at a given concentration. Xu et al. [20] further demonstrated in an ultrasonic cavitation field that even low aeration concentrations could effectively suppress cavitation erosion, with smaller bubbles outperforming larger bubbles at the same concentration. Although cumulative material mass loss decreased with increasing bubble population and air content, the mitigation effect eventually reached saturation. Overall, these studies indicate that aeration concentration is the dominant factor governing cavitation erosion mitigation, while air bubble size further modulates the mitigation performance at a given aeration concentration.

Alongside macroscopic studies on aeration-based cavitation mitigation, mesoscopic-scale mechanism research has also advanced. Under atmospheric pressure conditions, researchers have focused on cavitation bubble-air bubble interactions from bubble dynamics perspective to reveal the intrinsic mechanism of cavitation erosion mitigation. Smith and Mesler [21] first reported that an air bubble adjacent to a solid boundary could suppress cavitation erosion damage on the wall. Xu et al. [22] introduced dimensionless parameters such as relative size, bubble–bubble distance, bubble-wall distance, and spatial orientation. These parameters were used to quantify changes in the movement path and collapse direction of a wall-adjacent cavitation bubble caused by a nearby air bubble. Luo et al. [23] further examined how a free air bubble influences a cavitation bubble. They found that the neighboring air bubble significantly changed the evolution duration of cavitation bubbles and underwent noticeable deformation during the interaction. Goh et al. [24] investigated how an attached air bubble redirects the collapse-induced microjet from a near-wall cavitation bubble. Their results showed that the microjet direction can point either toward or away from the wall, depending on the geometric configuration. Luo et al. [25] further proposed that, when a nearby air bubble coexists with a solid boundary, the cavitation bubble collapse direction follows the vector sum of their effects. Gonzalez-Avila et al. [26] reported a biomimetic gas-entrapping microtextured surface (GEMS) that can stably retain air. The trapped air repels nearby cavitation bubbles away from the surface, thereby preventing cavitation erosion. This study provides an important strategy for cavitation erosion protection through surface microstructures. Agrež et al. [27] investigated the interaction between laser-induced cavitation bubbles and hydrogen bubbles attached to a flat electrode surface. They found that the vortical flow generated by cavitation bubble collapse could detach the hydrogen bubbles from the surface. This finding improves the theoretical understanding of the interaction between surface-attached gas bubbles and cavitation bubbles.

As research progressed, the focus shifted from qualitative observation of cavitation bubble collapse morphology to quantitative measurements of its collapse dynamics. Luo et al. [28] showed that air bubbles affect shockwaves generated during cavitation bubble collapse. This effect varies strongly with the relative spacing of the two bubbles and the relative air bubble size. When the two bubbles merge, both the shockwave energy and peak pressure are significantly reduced. In addition, numerical simulations of air bubbles-cavitation bubbles interactions [29], [30], [31], [32] and studies of cavitation bubble behavior near free surfaces [33], [34], [35], [36], [37] have been extensively conducted. These works have helped clarify the mechanism by which aeration mitigates cavitation erosion. Beyond examining the effects of air bubbles on cavitation bubble dynamics, establishing a direct relationship between single-bubble collapse and material erosion is also important for revealing the mechanisms of cavitation erosion. Mur et al. [38] generated controllable single acoustic cavitation bubbles in the ultrasonic field. They then correlated the bubble dynamics with the cavitation erosion patterns on the material surface. This study provides an important reference for explaining macroscopic cavitation erosion through single bubble experiments.

The above literature review shows that few studies on aeration-based cavitation erosion mitigation and its underlying mechanisms, particularly the interaction between air bubbles and cavitation bubbles, have considered the effect of ambient pressure. However, with the increasing development of hydropower projects at high altitudes and the expansion of cavitation-related applications under low ambient pressure, the influence of reduced ambient pressure remains poorly understood. In particular, its effects on the efficiency of aeration-based cavitation erosion mitigation in gas–liquid two-phase flows and on the mesoscale dynamics of cavitation bubble-air bubble interactions remain largely unexplored. Therefore, this study first investigates the efficiency of aeration-based cavitation erosion mitigation in an ultrasonic gas–liquid two-phase flow under different low ambient pressures at the macroscale. Mesoscale experiments on the interaction between air bubbles and cavitation bubbles are then conducted to provide a preliminary explanation of the physical mechanisms underlying the macroscale cavitation erosion mitigation behavior. These two aspects of the study improve the understanding of aeration-based cavitation erosion mitigation and its mechanisms under low ambient pressure.

## 2 Experimental methods

**Fig. 1:**
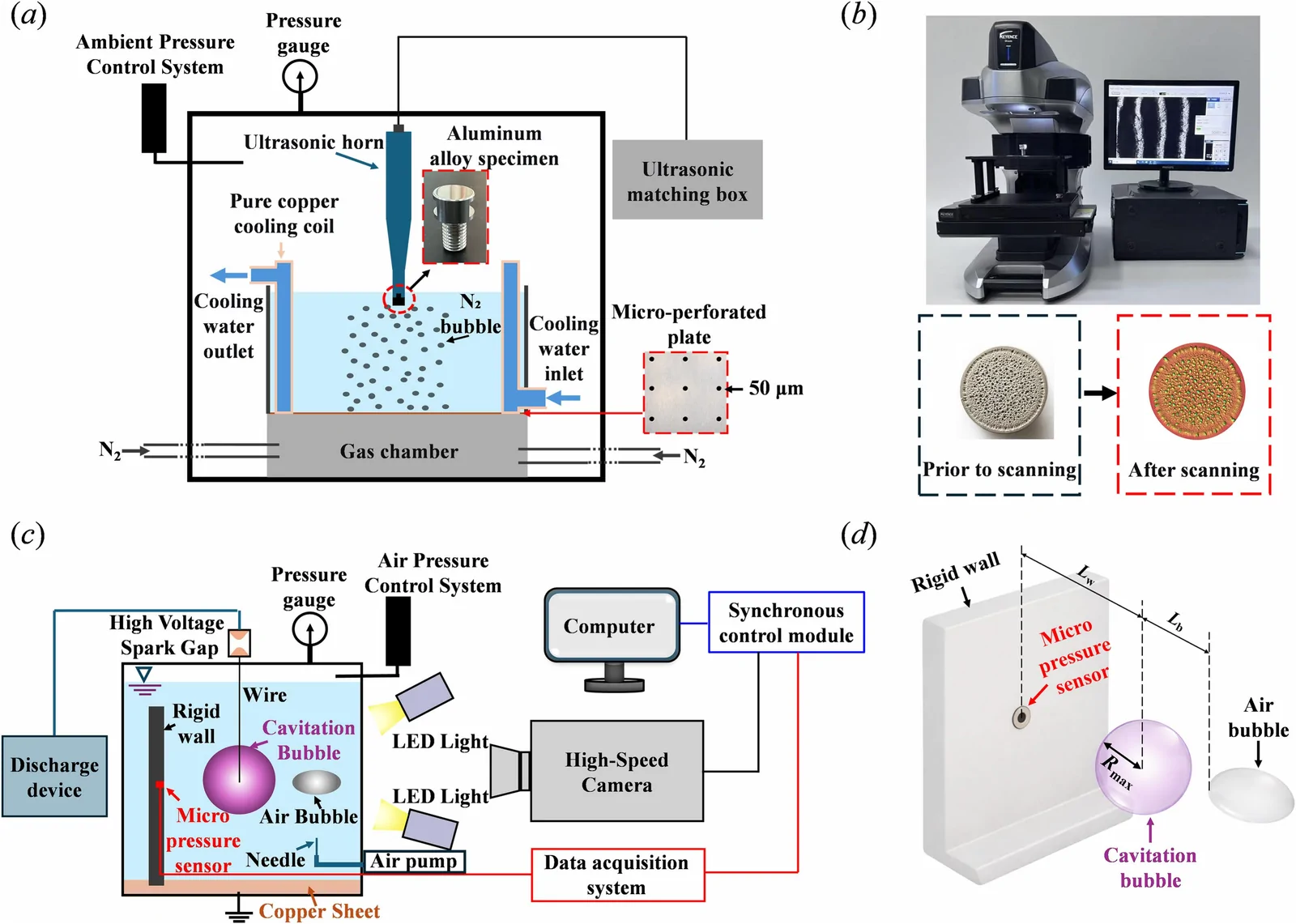
Experimental system configuration.

This study was conducted at both the macroscale and the mesoscale. At the macroscale, the effect of low ambient pressure (below 1.013 × 10^5^ Pa) on the efficiency of aeration-induced cavitation erosion mitigation was quantitatively investigated. At the mesoscale, the effects of air bubbles on the collapse characteristics of near-wall cavitation bubbles under different low ambient pressures were examined to explore the underlying mechanisms. For these purposes, an ultrasonic cavitation experimental system and a cavitation bubble dynamics experimental system were used, as shown in Fig. 1.

The experimental system for aeration-based cavitation erosion mitigation is presented in Fig. 1(a). The system was installed in a sealed chamber, where the ambient absolute pressure was adjusted from 55 to 95 kPa with a pressure control accuracy of ±0.2 kPa. Cavitation was generated by an ultrasonic vibrator (20 kHz, 110 W), a method widely used in cavitation studies [39], [40]. Specimens were made of 6061-T6 aluminum alloy and machined into bolted shapes according to ASTM G32-16 [41], then attached to the end of the ultrasonic horn. Entrained air bubbles were introduced from a water tank with a micro-perforated plate (pore size 50 µm) at the bottom: after the air chamber was pressurized, gas was uniformly released through the micro-perforated plate, forming a stable upward flow of entrained bubbles.

The aeration concentration near the horn tip was measured using a dual-needle conductivity probe [42]. The detailed measurement methods followed Refs. [42], [43]. In the gas–liquid two-phase flow, the raw voltage signal measured by the probe was converted into a binary instantaneous phase indicator sequence using a threshold criterion. Here, *c*_i_ = 0 represents the water phase, while *c*_i_ = 1 represents the gas phase. The local time-averaged air concentration (*C*) was defined as the fraction of the sampling period during which the gas phase occupied the measurement point. It was calculated as follows:

(1) $$C=\frac{1}{n}\sum_{i=1}^{n}c_{i}$$

where *n* is the total number of sampling points, calculated as the product of the sampling frequency and the sampling duration. *c*_i_ is the instantaneous phase indicator at sampling point *i*. In this experiment, the sampling frequency was set to 80 kHz, and the duration of each continuous sampling period was 40 s. For clarity, *C* is reported as a percentage in this study, and the aeration concentration was controlled by adjusting the air supply rate.

Since reduced ambient pressure promotes entrained bubble expansion, bubble size must be controlled under each ambient pressure condition to minimize the experimental error. In this study, the pore size of the micro-perforated plate was kept constant. The size of the air bubbles near the specimen surface was controlled by adjusting the distance between the micro-perforated plate and the specimen. During the adjustment, high-speed cameras are used to capture the entrained bubbles near the surface of the specimen. The equivalent diameter is calculated based on the projected area of the entrained bubbles in the high-speed photography images to determine their size. The distance between the plate and the specimen is adjusted using the aforementioned method under different ambient pressures to ensure that the size of entrained bubbles near the surface of the specimen remain essentially consistent, with a mean equivalent circle diameter of approximately 1.49 mm.

It should be noted that the micropores on the micro-perforated plate were uniformly arranged in an equally spaced array. The gas first entered the chamber beneath the plate and was then released through the micropores. This process generated a stable group of rising air bubbles. The effective aeration area of the plate was larger than the projected area of the specimen surface. In addition, the centers of the plate and the specimen were always coaxially aligned. Therefore, the specimen was fully covered by the bubble group. This arrangement reduced the influence of lateral spreading at the edge of the bubble group on the experimental results. Under different ambient pressures, the pore diameter, pore spacing, effective aeration area, horizontal position, and gas supply method remained unchanged. Only the vertical distance between the plate and the specimen was adjusted to maintain a similar air bubble size. The initial horizontal release positions and the effective coverage area of the air bubbles remained unchanged. Therefore, adjustment of this distance had very little effect on the horizontal distribution of the bubble group. Moreover, air concentration and air bubble size were measured directly at fixed positions near the specimen surface. These measured values were used to control the experimental conditions. This method ensured a relatively uniform distribution of air bubbles near the specimen and provided good comparability among the experiments conducted under different ambient pressures.

All experimental water was prepared from deionized water, boiled, and degassed under near-vacuum conditions for 72 h to reduce dissolved gas content and initial cavitation nuclei. To further reduce gas dissolution and help maintain comparable nuclei conditions at different ambient pressures, nitrogen was used as the aeration gas because of its chemical stability and low water solubility. Because temperature can affect ultrasonic cavitation erosion results [44], [45], the test water was kept at 25 ± 0.2°C throughout the experiments using a copper cooling coil.

After each cavitation erosion test, the specimen surface was optically scanned at 160× magnification using a 3D profilometer (KEYENCE VR-5000) to characterize the pit distribution, as presented in Fig. 1(b). In addition, the mass loss of each specimen was quantified. Before each cavitation erosion test, the specimen was weighed three times using an analytical balance (MS204, METTLER TOLEDO; resolution: 0.01 mg), and the mean of the three measurements was taken as its initial mass. After the test, the specimen was cleaned, thoroughly oven-dried, and allowed to cool to room temperature. It was then weighed three times using the same balance, and the mean value was taken as its post-test mass. The mass loss in each test was calculated as the difference between the mean specimen masses measured before and after cavitation exposure.

Fig. 1(c) presents the system used to investigate cavitation bubble dynamics. It comprised a corona discharge device for cavitation bubble generation, a high-frame-rate imaging system, and a miniature pressure sensor with high frequency response. The experiments were also conducted in the sealed chamber, with ambient pressure adjusted in the same manner. The corona discharge device was connected to the discharge electrode via a spark gap. The electrode tip, coated with insulation, had a diameter of approximately 0.3 mm. A copper plate connected to ground was installed at the chamber bottom to form a stable discharge circuit. By adjusting the discharge voltage and capacitor, the discharge energy could be precisely controlled, ensuring high repeatability of cavitation bubble size [46]. In all experiments of this study, the discharge voltage and capacitance are kept constant. The high-frame-rate imaging system (Photron FASTCAM SA-Z, maximum 1,000,000 fps) operated at 180,000 fps. Two lighting methods were used: a 350 W LED lamp and a 200 W flat-panel lamp for front lighting during microjet visualization, and a schlieren optical setup for shockwave visualization. Before each experiment, a calibration scale was placed in the imaging plane of the cavitation bubble to obtain a calibration image and establish the relationship between pixel dimensions and physical length. The time interval between successive images was determined from the camera frame rate. The position of the microjet tip was identified frame by frame from the high-speed images. Because the air bubble reduced the microjet velocity, the velocity was calculated from the total displacement of the jet tip over multiple consecutive frames divided by the corresponding total time interval. This approach reduced the influence of jet-tip localization errors in individual frames. The maximum value obtained from different multi-frame intervals was taken as an approximation of the maximum microjet velocity. Microjet morphology was qualitatively characterized in terms of its contour, tip shape, and continuity. The uncertainty in the microjet velocity measurements mainly arose from run-to-run differences in spatial calibration and errors in identifying the microjet-tip position.

Single air bubbles were generated near the cavitation bubble through a needle connected to an air pump. The inner diameter of the gas needle ranged from 0.06 to 7 mm. In the mesoscale experiments, the mean equivalent radius of the air bubbles ranged from approximately 1.85 to 6.72 mm. Under different ambient pressures, air bubbles with similar dimensionless sizes (*ε*) were generated by adjusting the inner diameter of the gas needle and the gas supply conditions. The straight-line distance between the gas needle tip and the centroid of the cavitation bubble was approximately 120 mm. This distance was more than nine times the maximum cavitation bubble radius under all tested ambient pressures. Therefore, the influence of the gas needle on the cavitation bubble dynamics could be considered negligible. A miniature pressure sensor with high frequency response (Test Electronics Information Co. Ltd, Measurement Range: 20 MPa; Accuracy: ±0.05 MPa; Maximum Frequency Bandwidth: 500 kHz; Risetime: 0.2 μs) was installed flush with the rigid wall surface to record the shockwave pressure as a wall-adjacent cavitation bubble collapsed. The evolution of cavitation bubbles is accompanied by expansion shockwaves, first-collapse shockwaves, and even secondary-collapse shockwaves. In this study, only the first-collapse shockwaves were measured. For each recorded pressure signal of the first-collapse shock wave, the maximum pressure was taken as the peak shockwave pressure. It should be noted that, due to the limited sampling frequency, this value may not represent the true peak pressure of the shock wave, but the difference between them is very small.

Fig. 1(d) presents the spatial arrangement among the solid wall, cavitation bubble, and neighboring air bubble. The geometric relationships among these components are described using three dimensionless parameters: *γ* (=*L*_w_/*R*_max_), the normalized bubble-wall stand-off distance; *ε* (=*r*/*R*_max_), representing the air bubble size normalized by the maximum cavitation bubble radius; and *δ* (=*L*_b_/*R*_max_), representing the normalized centroid to surface spacing for the cavitation bubble and air bubble. Here, *L*_w_ represents the actual bubble-wall spacing, *R*_max_ is the equivalent circular radius at cavitation bubble maximum expansion, *r* is the air bubble equivalent circular radius, and *L*_b_ represents the actual spacing separating the cavitation bubble centroid and the air bubble surface. It should be noted that the equivalent radius of both cavitation bubbles and air bubbles is calculated using the formula $R=\sqrt{A/\pi }$, where *A* represents the two-dimensional projected area of the cavitation bubble (or air bubble) in high-speed photography images.

To ensure the reliability of the experimental results, each test condition was independently repeated. The ultrasonic cavitation erosion experiments with aeration were repeated five times for each condition. In the mesoscale single cavitation bubble dynamics experiments, the near-wall cavitation bubble collapse experiments focusing on microjet were repeated five times for each condition, whereas those focusing on collapse-induced shockwaves were repeated four times. All quantitative results are reported as the mean of the repeated experiments, and the error bars represent the standard deviation.

## 3 Experimental observations

### 3.1 Effect of aeration concentration on cavitation erosion

**Fig. 2:**
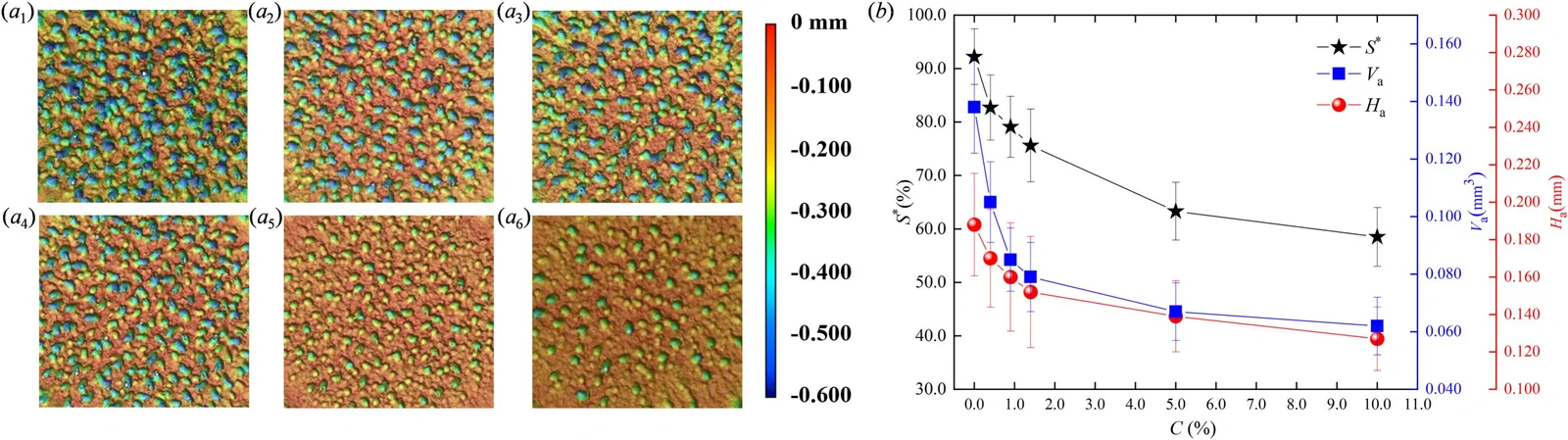
Cavitation erosion morphologies and characteristic erosion parameters of the specimens under different aeration concentrations at *P*_atm_ = 95 kPa and *T*_e_ = 90 min, where panels (*a*_1_)–(*a*_6_) correspond to *C* = 0%, 0.40%, 0.90%, 1.40%, 5.00%, and 10.00%, respectively.

Under constant ambient pressure (*P*_atm_), the variation in cavitation erosion with aeration concentration (*C*) was examined. Taking *P*_atm_ = 95 kPa as an example, Fig. 2 shows the three-dimensional cavitation erosion morphologies and characteristic erosion parameters of the aluminum alloy specimen after 90 min of cavitation exposure under different aeration concentrations.

In the absence of aeration (*C* = 0%), the specimen surface exhibited severe cavitation erosion damage, with noticeable merging of adjacent cavitation erosion pit edges (Fig. 2(*a*_1_)). The depth color map shows generally deep pits in the vertical direction, while extensive pit edge merging in the horizontal direction substantially increased the projected erosion area. After introducing entrained air bubbles (*C* = 0.40%), cavitation erosion damage on the specimen surface was markedly reduced (Fig. 2(*a*_2_)): pit edge merging decreased, pit depth decreased, and the projected area contracted accordingly. As the aeration concentration further increased to 0.90%, 1.40%, 5.00%, and 10.00%, the cavitation erosion damage continuously alleviated, as shown in Fig. 2(*a*_3_)–(*a*_6_).

To complement the qualitative observations of cavitation erosion morphology at different air concentrations, Fig. 2(b) provides a quantitative comparison of the surface damage after 90 min of cavitation exposure at *P*_atm_ = 95 kPa. Three cavitation erosion parameters were evaluated: the eroded-area ratio (S* = *S*_e_/*S* × 100%), the mean pit volume (*V*_a_) and the mean of the individual pit average depths (*H*_a_). Here, *S*_e_ denotes the surface area damaged by cavitation erosion and *S* is the area exposed to ultrasonic cavitation. As the air concentration *C* increased from 0% to 10%, S* decreased from 92.2% to 58.5%. Over the same range, *V*_a_ decreased from 0.138 to 0.062 mm^3^, while *H*_a_ decreased from 0.188 to 0.127 mm. All three parameters decreased steadily with increasing air concentration, indicating a progressive reduction in cavitation erosion.

It is worth noting that, unlike hydrodynamic cavitation [47], cavitation erosion was not completely suppressed in the present ultrasonic system, even at aeration concentrations of 5.00% and 10.00%. This difference may arise because the region near the specimen is continuously subjected to periodic acoustic pressure. Although air bubbles can weaken cavitation bubble collapse, the sustained ultrasonic field provides a continuous driving force. Cavitation bubbles can therefore undergo repeated growth and collapse cycles [48]. In contrast, hydrodynamic cavitation is induced by local pressure drops in the flow. Aeration not only cushions cavitation bubble collapse but may also alter the local flow structure and pressure field. These effects can limit the development of the cavitation zone, reduce violent near-wall cavitation bubble collapse and thereby mitigate the resulting cavitation erosion [49], [50]. Nevertheless, compared with the non-aerated condition, the extent of cavitation erosion induced by ultrasonic cavitation was significantly reduced at high aeration concentrations. As presented in Fig. 2(*a*_6_), at *C* = 10.00%, adjacent cavitation erosion pits rarely coalesced on the specimen surface. Most cavitation erosion pits were isolated, and their number was markedly reduced. Compared with the non-aerated condition, S*, *V*_a_ and *H*_a_ were all significantly reduced, indicating a pronounced reduction in cavitation erosion damage.

The above experimental results show that, at a given ambient pressure, cavitation erosion progressively decreased with increasing air concentration. Quantitatively, the eroded-area ratio, mean pit volume, and cluster-averaged mean pit depth all decreased. Qualitatively, the pit contours in the scanned images enclosed progressively smaller projected areas.

### 3.2 Effect of ambient pressure on cavitation erosion

**Fig. 3:**
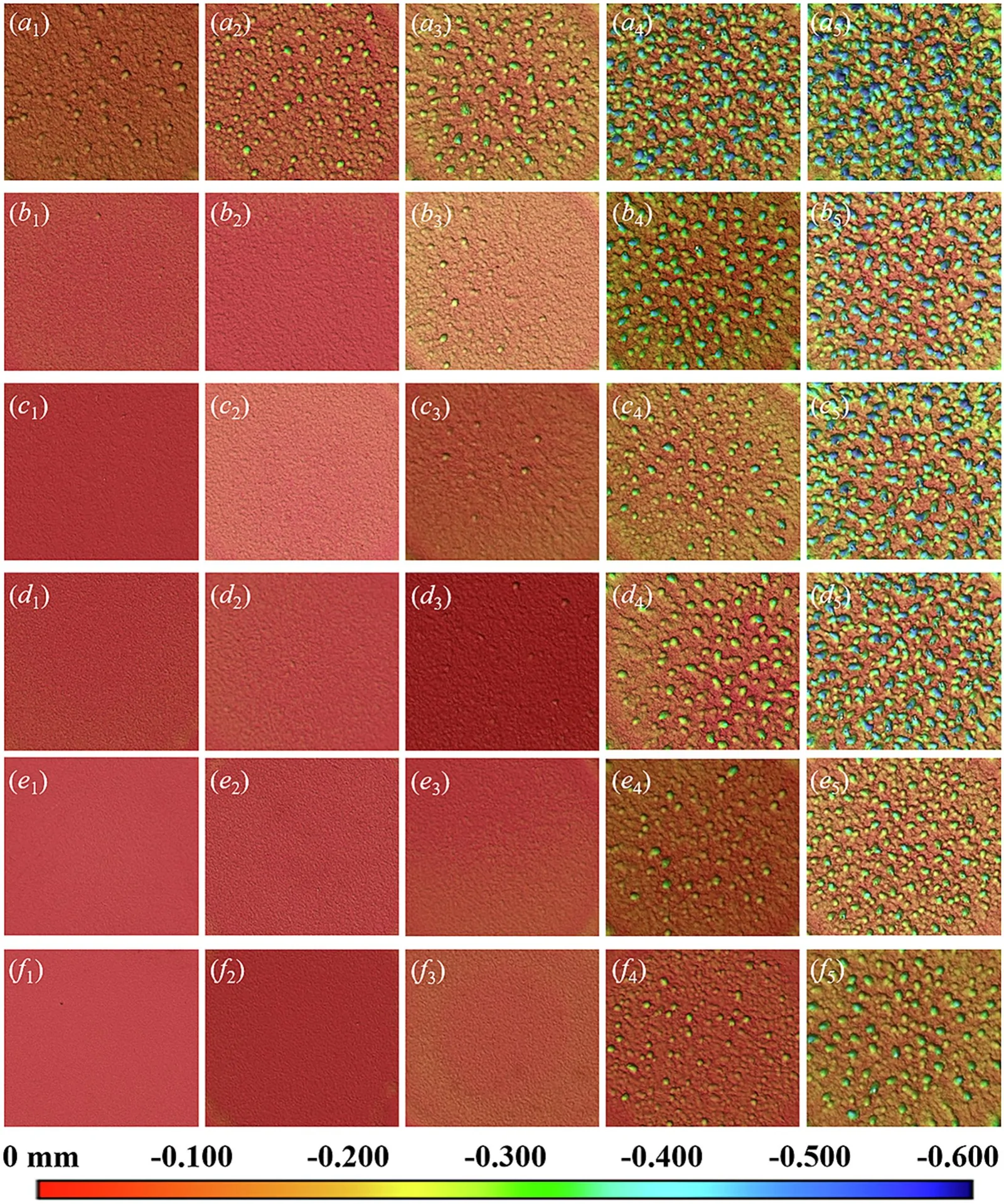
Evolution of cavitation erosion morphologies of the specimens with ambient pressure under the same aeration concentration at *T*_e_ = 90 min. Panels (*a*)-(*f*) correspond to *C* = 0%, 0.40%, 0.90%, 1.40%, 5.00%, and 10.00%, respectively. Labels 1–5 in each panel indicate *P*_atm_ = 55, 65, 75, 85, and 95 kPa, respectively.

The three-dimensional cavitation erosion morphologies of the aluminum alloy specimens after 90 min of cavitation exposure under different ambient pressures and aeration concentrations are presented in Fig. 3. At each ambient pressure, cavitation erosion damage was progressively weakened as aeration concentration increased, with a smaller pit depth and a smaller projected area of the pit contours. These results indicate that aeration can still effectively suppress cavitation erosion after a reduction in ambient pressure. At a given aeration concentration, cavitation erosion damage changed markedly as ambient pressure decreased.

Fig. 3(*a*_1_)–(*a*_5_) present the cavitation eroded surface morphologies of the specimens under the non-aerated condition (*C* = 0%). As *P*_atm_ decreased successively from 95 kPa to 85, 75, 65 and 55 kPa, the severity of cavitation erosion gradually decreased. Specifically, coalescence between the edges of adjacent cavitation erosion pits became less pronounced, the pits tended to become more isolated, and both the pit depth and the projected area of the pit contours continuously decreased. Taking the comparison between *P*_atm_ = 95 kPa and 55 kPa as an example (Fig. 3(*a*_5_) and (*a*_1_)), dense cavitation erosion pits appeared on the specimen surface at *P*_atm_ = 95 kPa. Extensive coalescence occurred between adjacent pit edges and the pits exhibited relatively large depths. In contrast, at *P*_atm_ = 55 kPa, fewer cavitation erosion pits were observed after the same exposure duration, with substantial decreases in pit depth and lateral expansion scale.

After the introduction of air bubbles, the cavitation erosion mitigation effect exhibited pronounced differences with ambient pressure at the same aeration concentration. Taking *C* = 0.40% as an example, at *P*_atm_ = 95 kPa, compared with *C* = 0%, coalescence between the edges of adjacent cavitation erosion pits was reduced, and both the pit depth and the projected area of the pit contours decreased. However, cavitation erosion on the specimen surface remained relatively pronounced, suggesting limited mitigation by the entrained air bubbles under this condition (Fig. 3(*a*_5_) and (*b*_5_)). The experimental observations at *P*_atm_ = 85 kPa were similar to those at *P*_atm_ = 95 kPa, although the cavitation erosion mitigation effect was slightly enhanced (Fig. 3(*a*_4_) and (*b*_4_)). When *P*_atm_ was further reduced to 75, 65, and 55 kPa, the cavitation erosion damage was substantially alleviated compared with that under the non-aerated condition at the corresponding pressure. For example, at *P*_atm_ = 55 kPa and *C* = 0.40%, after 90 min of cavitation exposure, only a very small number of isolated cavitation erosion pits could be identified on the specimen surface, both the pit depth and the projected area of the pit contours were significantly reduced (Fig. 3(*a*_1_) and (*b*_1_)). A lateral comparison further shows that, at the same aeration concentration, the severity of cavitation erosion continuously decreased as *P*_atm_ was successively reduced from 95 to 55 kPa (Fig. 3(*b*_1_)–(*b*_5_)). When *C* was further increased to 10.00%, cavitation erosion on the specimen surface was markedly mitigated under all ambient pressure conditions compared with the non-aerated condition, as shown by the comparison between Fig. 3(a) and (f). At *P*_atm_ = 95 and 85 kPa, visible cavitation erosion pits were still present on the specimen surface. In contrast, when *P*_atm_ was reduced to 55 kPa, no obvious cavitation erosion pits appeared after 90 min of exposure.

**Fig. 4:**
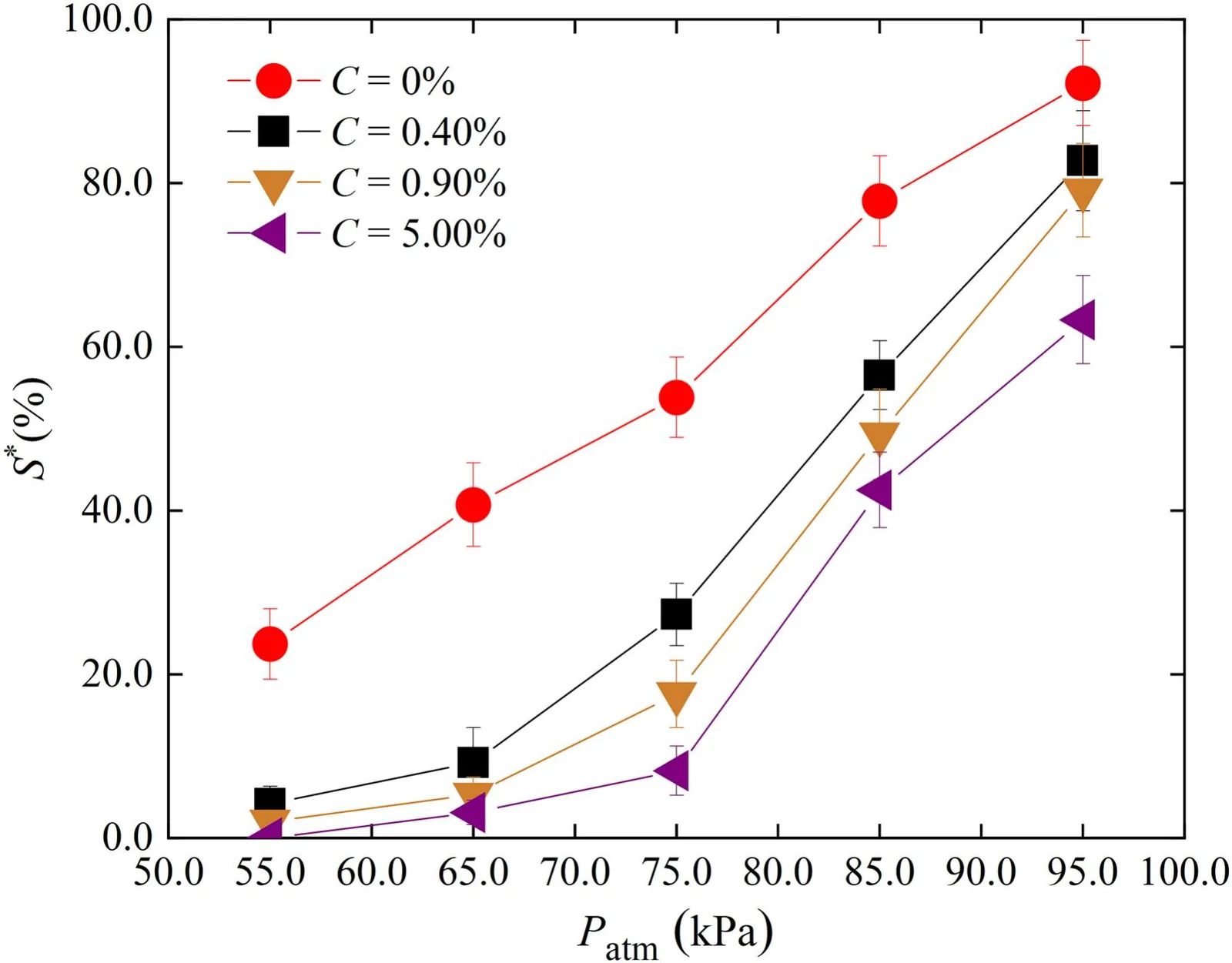
Variation of eroded-area ratio with *P*_atm_ at different air concentrations.

Fig. 4 further shows how the eroded-area ratio (S*) varied with *P*_atm_ after 90 min of cavitation exposure at different air concentrations. At any given *P*_atm_, S* decreased progressively as *C* increased. At *P*_atm_ = 95 kPa, increasing *C* from 0% to 0.40% reduced S* from 92.2% to 82.7%, corresponding to a relative reduction of approximately 10.3%. At *P*_atm_ = 55 kPa, the same increase in *C* reduced S* from 23.7% to 4.2%, corresponding to a relative reduction of approximately 82.3%. This reduction was substantially greater than that observed at *P*_atm_ = 95 kPa.

The above experimental results demonstrate that entrained air bubbles can still effectively suppress cavitation erosion after a reduction in ambient pressure and that higher aeration concentrations consistently lead to weaker cavitation erosion damage across all ambient pressures. Meanwhile, the cavitation erosion mitigation performance of entrained air bubbles is strongly affected by ambient pressure. Under nearly identical aeration conditions, the mitigation effect of entrained air bubbles becomes more pronounced as the ambient pressure decreases. Section 4 further quantifies how cavitation erosion pits evolve and how target mass loss changes under different ambient pressures and aeration concentration. It also systematically analyzes the variation in the cavitation erosion mitigation efficiency of entrained air bubbles with ambient pressure.

## 4 Macroscopic trend analysis

### 4.1 Cavitation erosion pit characteristics

**Fig. 5:**
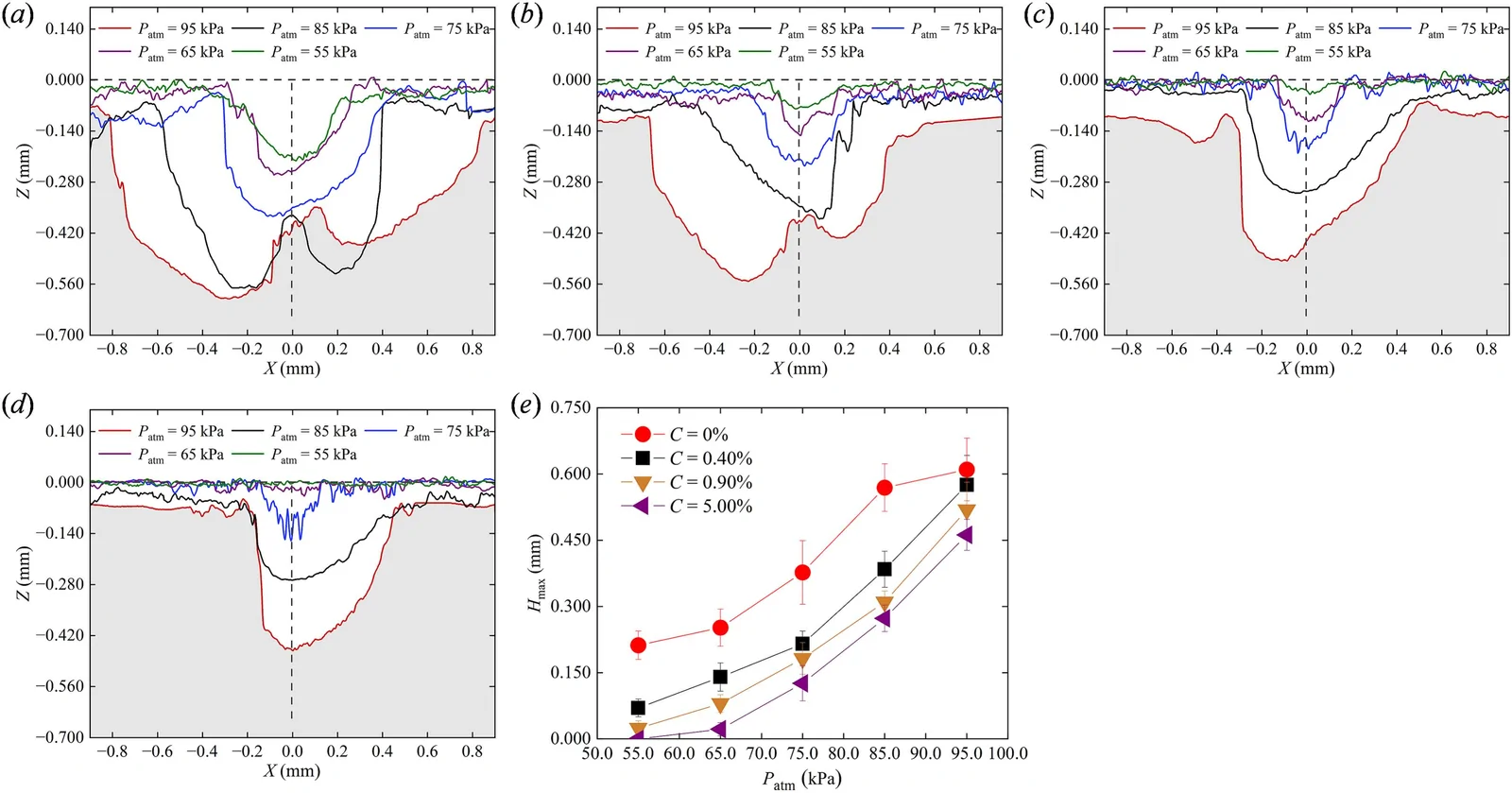
Variation in cavitation erosion pit profiles and mean value of the maximum pit depth with ambient pressure under different aeration concentrations at *T*_e_ = 90 min. Panels (*a*)-(*d*) correspond to *C* = 0%, 0.40%, 0.90%, 5.00%, respectively; Panel (*e*) shows the variation in the mean maximum pit depth.

After the 90 min of cavitation exposure, the deepest cavitation erosion pit within the pit cluster was selected and a longitudinal section was taken along the major axis of its planar projected contour to obtain the longitudinal profile curve of the pit. Fig. 5 presents the longitudinal profiles of the deepest cavitation erosion pits at different ambient pressures (*P*_atm_) and aeration concentrations (*C*), together with the variation in the mean value of maximum pit depth with *P*_atm_, as determined from five independent replicate experiments under each test condition. It should be noted that each profile curve in Fig. 5(a)–(d) was extracted from the single deepest pit under the corresponding condition, characterizing the geometry of the most severe local damage.

Fig. 5(a) compares the profile curves of representative cavitation erosion pits at different *P*_atm_ values under the non-aerated condition (*C* = 0%). As can be observed, the maximum pit depth, *H*_max_, defined as the absolute value of the maximum downward depth in the pit profile, decreased significantly with decreasing *P*_atm_. At *P*_atm_ = 95 kPa, *H*_max_ was 0.601 mm. When *P*_atm_ was successively reduced to 85, 75, 65, and 55 kPa, *H*_max_ decreased to approximately 0.570, 0.374, 0.260, and 0.221 mm, corresponding to reductions of approximately 5.2%, 37.8%, 56.7%, and 63.2%, respectively, relative to that at *P*_atm_ = 95 kPa. In addition, the pit diameter also decreased as *P*_atm_ decreased. These results indicate that, under the non-aerated condition, the severity of cavitation erosion continuously weakened with decreasing ambient pressure.

A comparison of *H*_max_ at the same ambient pressure but under different aeration concentrations (Fig. 5(a)–(d)) shows that *H*_max_ decreased with increasing *C* under all *P*_atm_ conditions. At *P*_atm_ = 95 kPa, as *C* increased successively from 0% to 0.40%, 0.90%, and 5.00%, *H*_max_ was 0.601, 0.552, 0.496, and 0.461 mm, respectively, corresponding to reductions of approximately 8.2%, 17.5%, and 23.3% relative to that at *C* = 0%. Notably, as *P*_atm_ decreased, the relative reduction in *H*_max_ at the same *C* increased markedly. At *P*_atm_ = 55 kPa, as *C* increased successively from 0% to 0.40%, 0.90%, and 5.00%, *H*_max_ was 0.221, 0.071, 0.039, and 0 mm, respectively, corresponding to reductions of approximately 67.9%, 82.4%, and 100.0% compared with the non-aerated condition at the same ambient pressure. Fig. 5(a)–(d) show the geometry of the deepest cavitation erosion pits under different test conditions and illustrate how their profiles changed with ambient pressure. Because a single profile cannot capture the variation among repeated tests, Fig. 5(e) further presents the mean value of *H*_max_ obtained from five independent tests for each condition as a function of ambient pressure. This comparison was included to verify the reproducibility of the observed trend in *H*_max_. Without aeration, *H*_max_ decreased markedly as the ambient pressure decreased. At a given ambient pressure, it also decreased progressively with increasing air concentration. Moreover, the reduction was more pronounced at lower ambient pressures.

These quantitative results indicate that, under the same air bubble size and aeration concentration conditions, aeration becomes more effective in mitigating cavitation erosion after ambient pressure is reduced.

**Fig. 6:**
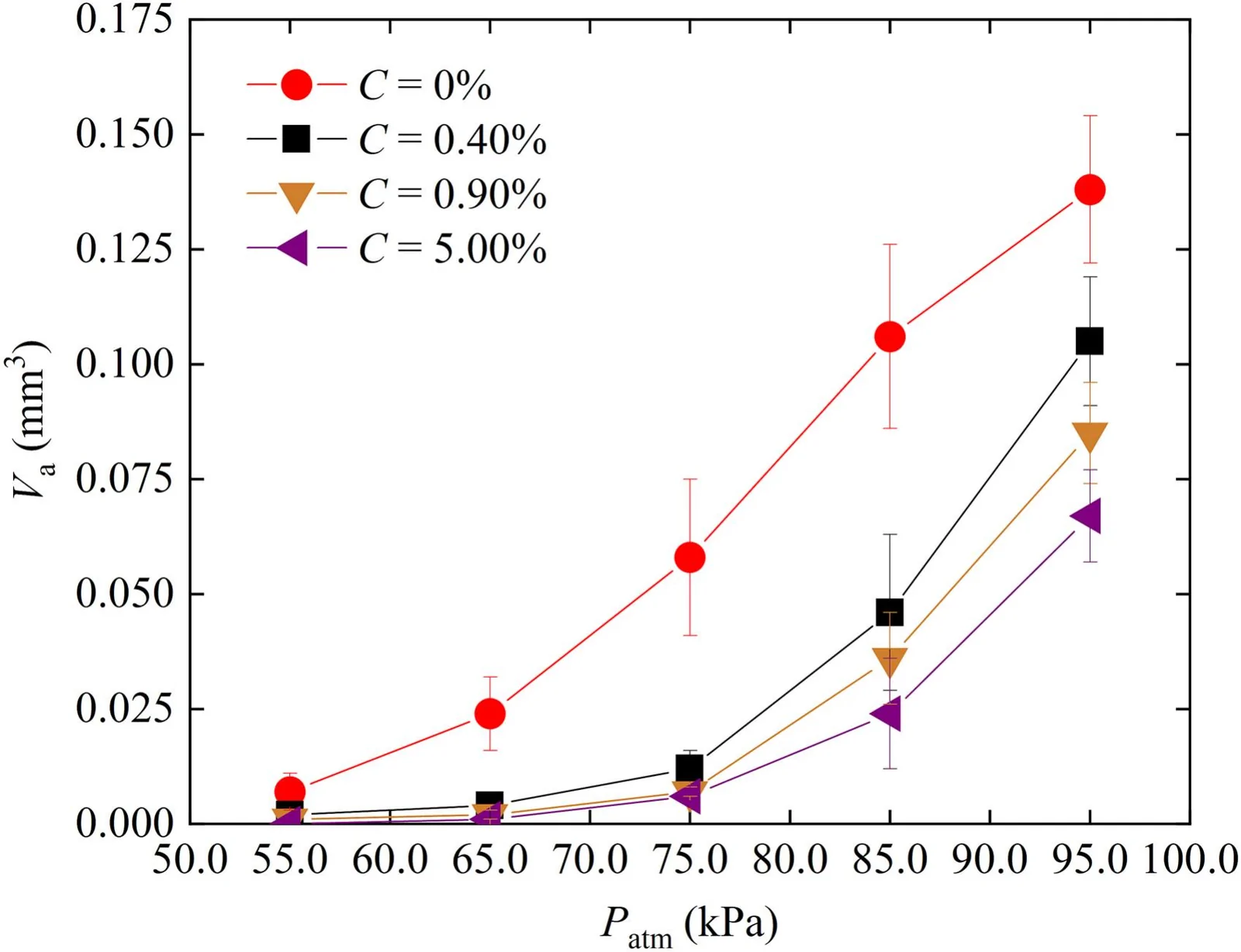
Variation of the mean cavitation erosion pit volume with ambient pressure at different air concentrations.

Fig. 6 shows the variation in the mean value of the cavitation erosion pit volume (*V*_a_) with *P*_atm_ after the aluminum alloy specimens were subjected to cavitation exposure for 90 min at different air concentrations. At a given *P*_atm_, *V*_a_ decreased progressively with increasing *C*. Similarly, at a given *C*, *V*_a_ decreased as *P*_atm_ decreased. For example, when *C* increased from 0% to 0.40%, *V*_a_ decreased from 0.138 to 0.105 mm^3^ at *P*_atm_ = 95 kPa, corresponding to a reduction of approximately 23.9%. At *P*_atm_ = 55 kPa, *V*_a_ decreased from 0.007 to 0.002 mm^3^, corresponding to a reduction of approximately 71.4%. This reduction was substantially greater than that observed at *P*_atm_ = 95 kPa, indicating that the cavitation erosion mitigation efficiency of aeration increased as the ambient pressure decreased.

In summary, ambient pressure has a significant influence on both cavitation erosion damage and aeration-based cavitation erosion mitigation efficiency. Under non-aerated conditions, the maximum depth and mean volume of the cavitation erosion pits decreased markedly as ambient pressure declined when the cavitation conditions remained the same. This indicates that cavitation erosion became weaker as ambient pressure decreased. Under nearly identical air bubble size, aeration concentration, and cavitation conditions, the maximum pit depth and mean pit volume also decreased with decreasing ambient pressure. Moreover, the reduction efficiency of both parameters due to entrained air bubbles gradually increased as ambient pressure decreased, indicating that the cavitation erosion mitigation efficiency of entrained air bubbles was enhanced under lower ambient pressure.

### 4.2 Mass loss characteristics

**Fig. 7:**
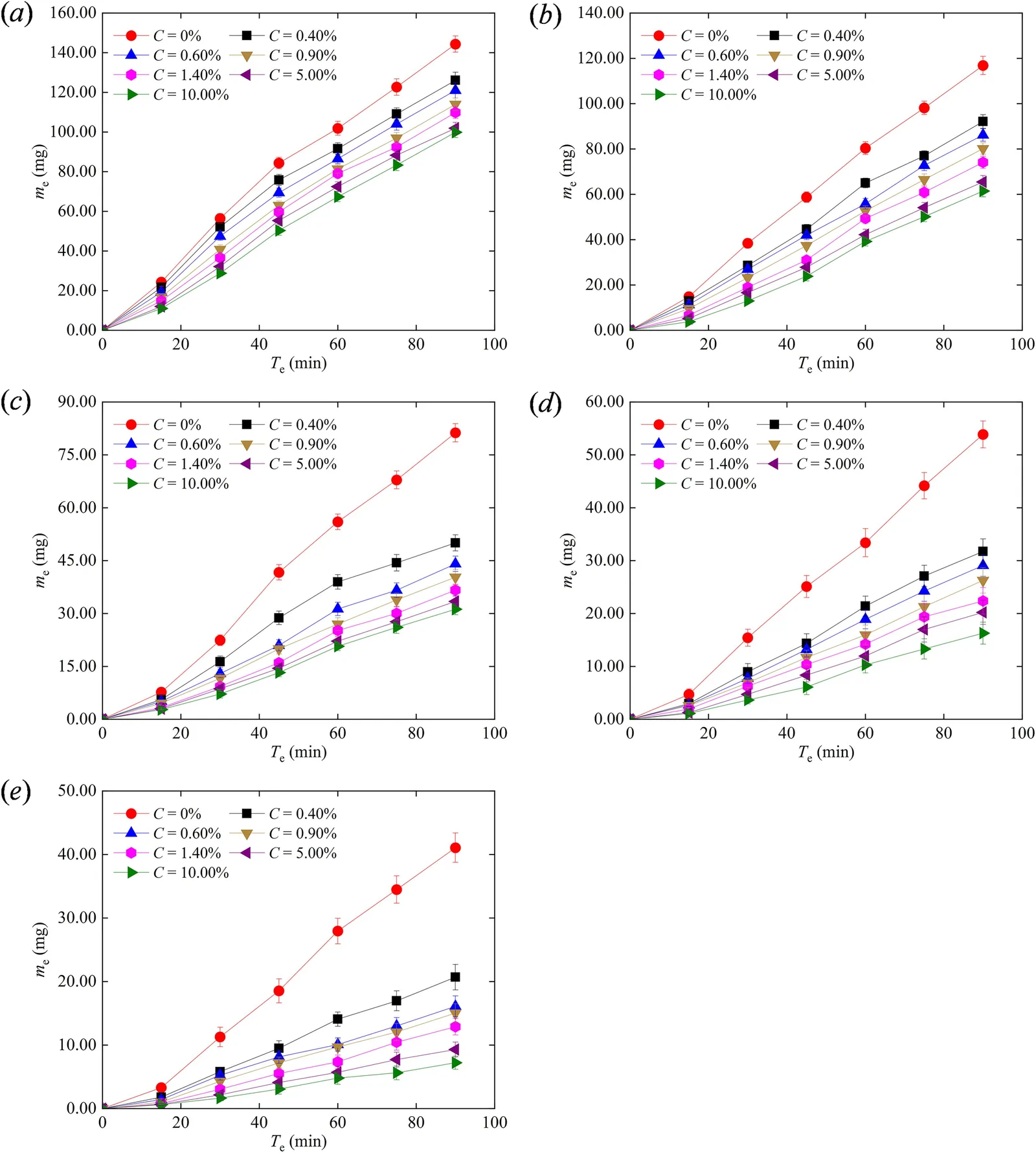
Time evolution of accumulated mass loss under different aeration concentrations and ambient pressures. Panels (*a*)-(*e*) correspond to *P*_atm_ = 95, 85, 75, 65 and 55 kPa.

The accumulated mass loss of the specimen is widely used to assess cavitation erosion damage [51], [52]. This section analyzes how the cumulative mass loss of aluminum alloy specimens (*m*_e_) evolves with cavitation exposure time (*T*_e_) under different ambient pressures and aeration concentrations. This aims to further clarify the role of ambient pressure in cavitation erosion mitigation by entrained air bubbles. The variation in *m*_e_ with *T*_e_ under different ambient pressures (*P*_atm_) and aeration concentrations (*C*), is presented in Fig. 7. The standard deviations across the different test conditions ranged from approximately 0.15 to 4.12 mg.

As shown in Fig. 7, under all ambient pressure and aeration concentration conditions, *m*_e_ continuously increased with increasing *T*_e_. For *P*_atm_ = 55–95 kPa, the introduction of air bubbles significantly reduced *m*_e_ at each *T*_e_ compared with the non-aerated condition. When *T*_e_ was relatively short (*T*_e_ = 15 min), the differences in *m*_e_ among different *C* conditions were relatively small. As *T*_e_ increased, these differences became progressively more pronounced. Overall, at a given *P*_atm_ and *T*_e_, *m*_e_ continuously decreased with increasing *C* (Fig. 7(a)). Taking *P*_atm_ = 95 kPa and *T*_e_ = 90 min as an example, relative to the non-aerated condition (*C* = 0%), *m*_e_ decreased by approximately 12.6%, 16.1%, 21.0%, 23.9%, 29.4%, and 30.8% at *C* = 0.40%, 0.60%, 0.90%, 1.40%, 5.00%, and 10.00%, respectively.

A comparison of Fig. 7(a)–(d) shows that ambient pressure strongly affects specimen cumulative mass loss in both non-aerated and aerated cases. From the perspective of extreme value comparison, at *P*_atm_ = 95 kPa *m*_e_ was approximately 144.31 mg and 99.89 mg at *C* = 0% and *C* = 10.00%, respectively. In contrast, at *P*_atm_ = 55 kPa, the corresponding values were approximately 44.37 mg and 7.22 mg, respectively. Under the non-aerated condition, *m*_e_ at *P*_atm_ = 95 kPa was approximately 3.3 times that at *P*_atm_ = 55 kPa. At *C* = 10.00%, this ratio increased to approximately 13.8. These results show that lower ambient pressure leads to reduced cumulative mass loss and significantly improved cavitation erosion mitigation efficiency of entrained air bubbles.

**Fig. 8:**
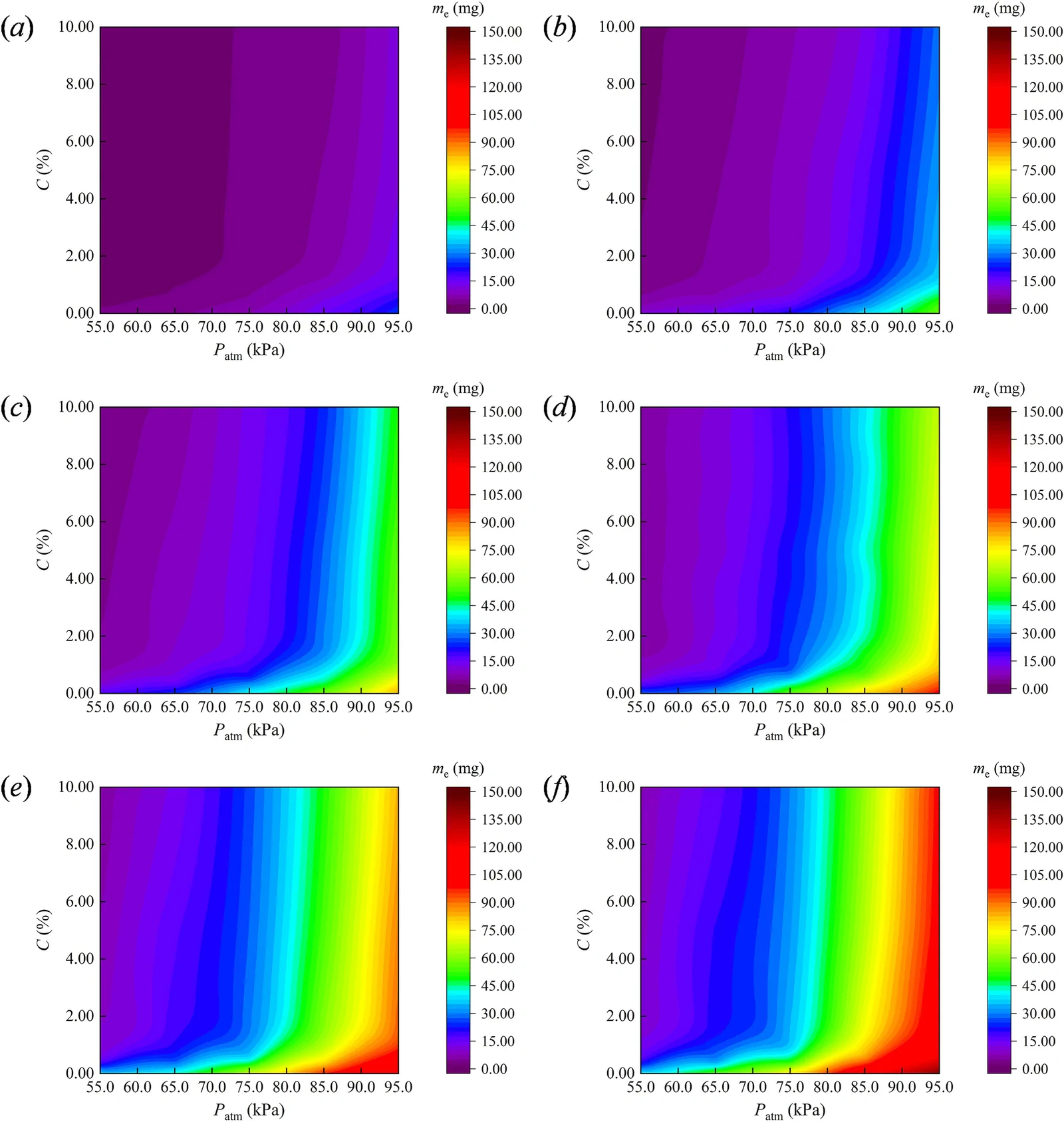
Contour maps of cumulative mass loss under varying ambient pressures and aeration concentrations. Panels (*a*)-(*f*) correspond to *T*_e_ = 15, 30, 45, 60, 75 and 90 min.

To further reveal the synergistic reduction effect of ambient pressure and aeration concentration in reducing specimen mass loss, *m*_e_ was analyzed over the full parameter range of *P*_atm_ = 55–95 kPa and *C* = 0%-10%. The results are presented as a two-dimensional contour map in Fig. 8.

As shown in Fig. 8, *m*_e_ increased to varying degrees with increasing *T*_e_ under all ambient pressure and aeration concentration conditions. At *T*_e_ = 15 min, the values of *m*_e_ were generally small under different *P*_atm_ and *C* conditions, and the differences among them were not significant (Fig. 8(a)). As *T*_e_ increased, *m*_e_ continued to increase, while the differences among different conditions gradually enlarged. Fig. 8(f) shows the variation of *m*_e_ with *P*_atm_ and *C* at *T*_e_ = 90 min. As can be observed, lower ambient pressure and higher aeration concentration resulted in smaller *m*_e_: at a given *P*_atm_, *m*_e_ decreased with increasing *C*, whereas at a given *C*, *m*_e_ decreased with decreasing *P*_atm_. In terms of the synergistic reduction effect of ambient pressure and aeration concentration on *m*_e_, when the value of *m*_e_ at *P*_atm_ = 95 kPa and *C* = 0% was used as the reference, entrained air bubbles reduced *m*_e_ by approximately 95.0% as *P*_atm_ decreased to 55 kPa and *C* increased to 10.00%.

### 4.3 Cavitation erosion rate characteristics

This section further investigates how the mass erosion rate of aluminum alloy specimens changes with ambient pressure (*P*_atm_) and aeration concentration (*C*). The erosion rate was defined as the cumulative mass loss over a given exposure period divided by the corresponding cavitation exposure time:

(2) $$v_{e}=\frac{m_{e}}{T_{e}}$$

where *m*_e_ is the cumulative mass loss of the specimen over a given cavitation exposure period, and *T*_e_ is the cavitation exposure time.

**Fig. 9:**
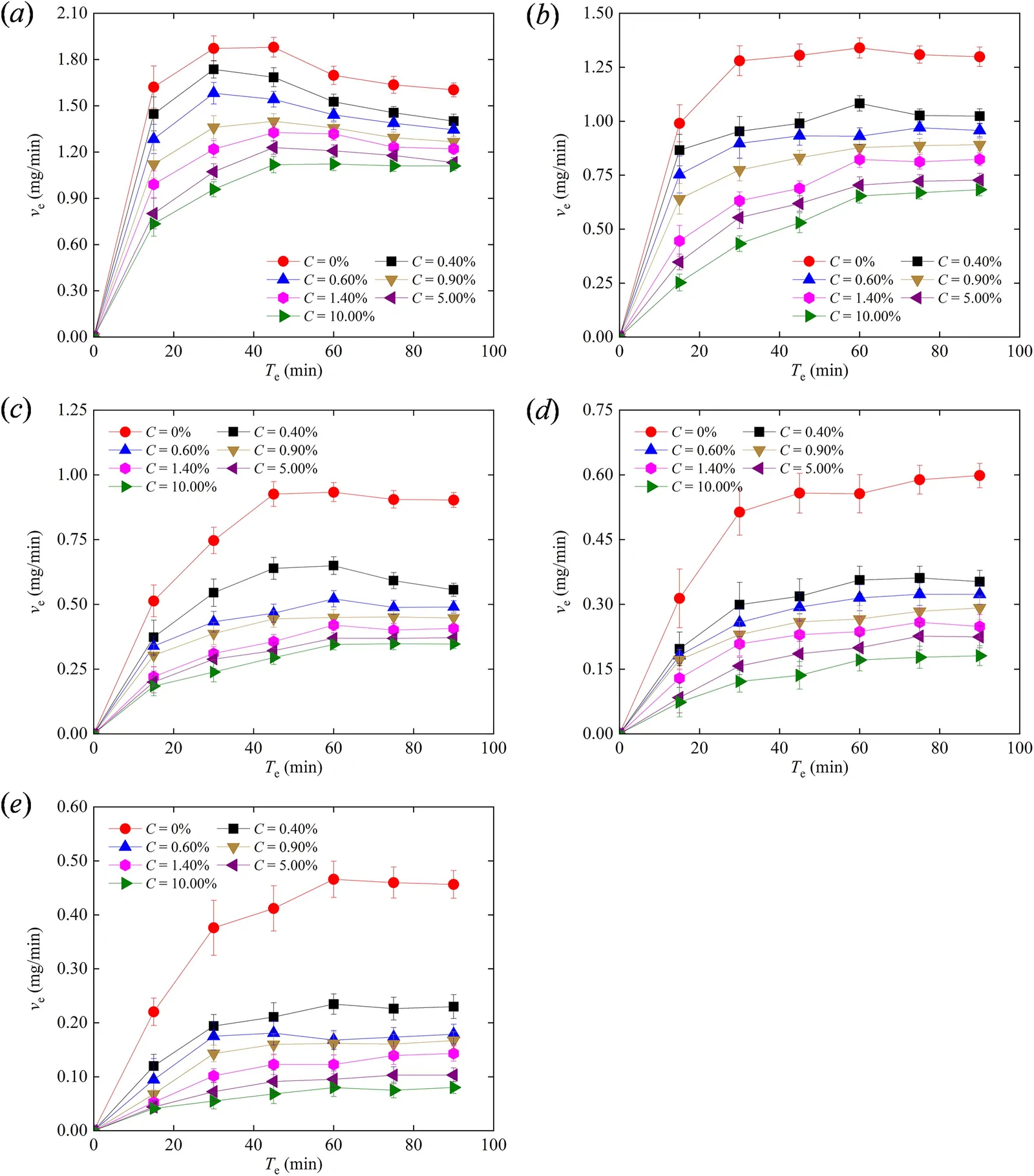
Time evolution of the mass erosion rate at different ambient pressures and aeration concentrations. Panels (*a*)-(*e*) correspond to *P*_atm_ = 95, 85, 75, 65 and 55 kPa.

Fig. 9 shows the variation in the mass erosion rate (*v*_e_) over cavitation exposure time (*T*_e_) at different *P*_atm_ and *C* values.

Fig. 9(a) shows the evolution of *v*_e_ with *T*_e_ under varying aeration concentrations at *P*_atm_ = 95 kPa. As can be observed, when *C* = 0%, the cavitation erosion process of the specimen exhibited distinct acceleration, peak, deceleration, and steady stages. As *C* increased, *v*_e_ showed an overall decreasing trend. Taking *T*_e_ = 90 min as an example, *v*_e_ was 1.60 mg/min at *C* = 0%, whereas it decreased significantly to 1.11 mg/min when *C* increased to 10.00%. In addition, with increasing *C*, the stage-wise characteristics of *v*_e_ during its evolution with *T*_e_ gradually became less pronounced. In particular, the deceleration stage became less evident, and *v*_e_ entered the steady stage directly after reaching its peak. This behavior may result from the attenuation of cavitation bubble collapse intensity by entrained air bubbles.

As presented in Fig. 9(b)–(e), following the reduction in ambient pressure, the evolution of *v*_e_ with *T*_e_ under different aeration concentrations was generally similar to that observed at *P*_atm_ = 95 kPa. Specifically, at a given *P*_atm_ and *T*_e_, *v*_e_ decreased with increasing *C*. However, both the magnitude of *v*_e_ and its stage-wise evolution exhibited varying degrees of difference. A comparison of Fig. 9(a)–(e) shows that *v*_e_ decreased significantly at each *T*_e_ as *P*_atm_ decreased, and that the relative reduction in *v*_e_ at the same aeration concentration was more pronounced under lower ambient pressure than under higher ambient pressure. Taking *T*_e_ = 90 min as an example, at *P*_atm_ = 95 kPa and *C* = 10.00%, *v*_e_ was approximately 1.11 mg/min, which was approximately 30.6% lower than that at *C* = 0%. Whereas at *P*_atm_ = 55 kPa and *C* = 10.00%, *v*_e_ was approximately 0.08 mg/min, showing an 81.7% decrease compared with the non-aerated condition at the same ambient pressure. These results indicate that, under nearly identical cavitation and aeration conditions, reducing ambient pressure results in a lower *v*_e_ and a higher cavitation erosion mitigation efficiency of entrained air bubbles. In addition, as ambient pressure decreased, the stage-wise characteristics of the *v*_e_-*T*_e_ evolution became significantly less pronounced. This is likely attributable to the fact that cavitation bubble collapse intensity is already attenuated under reduced ambient pressure, and the additional suppression of near-wall bubble collapse by entrained air bubbles further diminishes *v*_e_, causing the stage-wise features to gradually disappear.

### 4.4 Cavitation erosion mitigation efficiency of entrained air bubbles

The experimental results presented in 4.1, 4.2, 4.3 show that, under nearly identical cavitation conditions, air bubble size, and aeration concentration, the eroded-area ratio, pit depth, mean pit volume, cumulative mass loss, and mass erosion rate all decreased with decreasing ambient pressure. Moreover, at the nearly identical aeration concentration, the reductions in these erosion parameters relative to the non-aerated condition increased as ambient pressure decreased, indicating that the aeration-based cavitation erosion mitigation efficiency was enhanced under lower ambient pressure. This section further quantitatively analyzes the variation in the cavitation erosion mitigation efficiency of entrained air bubbles under decreasing ambient pressure based on specimen cumulative mass loss.

The cavitation erosion mitigation efficiency of aeration, calculated from the mass loss of aluminum alloy specimens, is defined as follows:

(3) $$\eta_{m}=\frac{m_{e,0}-m_{e,air}}{m_{e,0}}\times 100\%$$

where *m*_e,0_ is the cumulative mass loss under the non-aerated condition at a given cavitation exposure time and ambient pressure, and *m*_e,air_ is the cumulative mass loss with aeration under otherwise identical conditions.

**Fig. 10:**
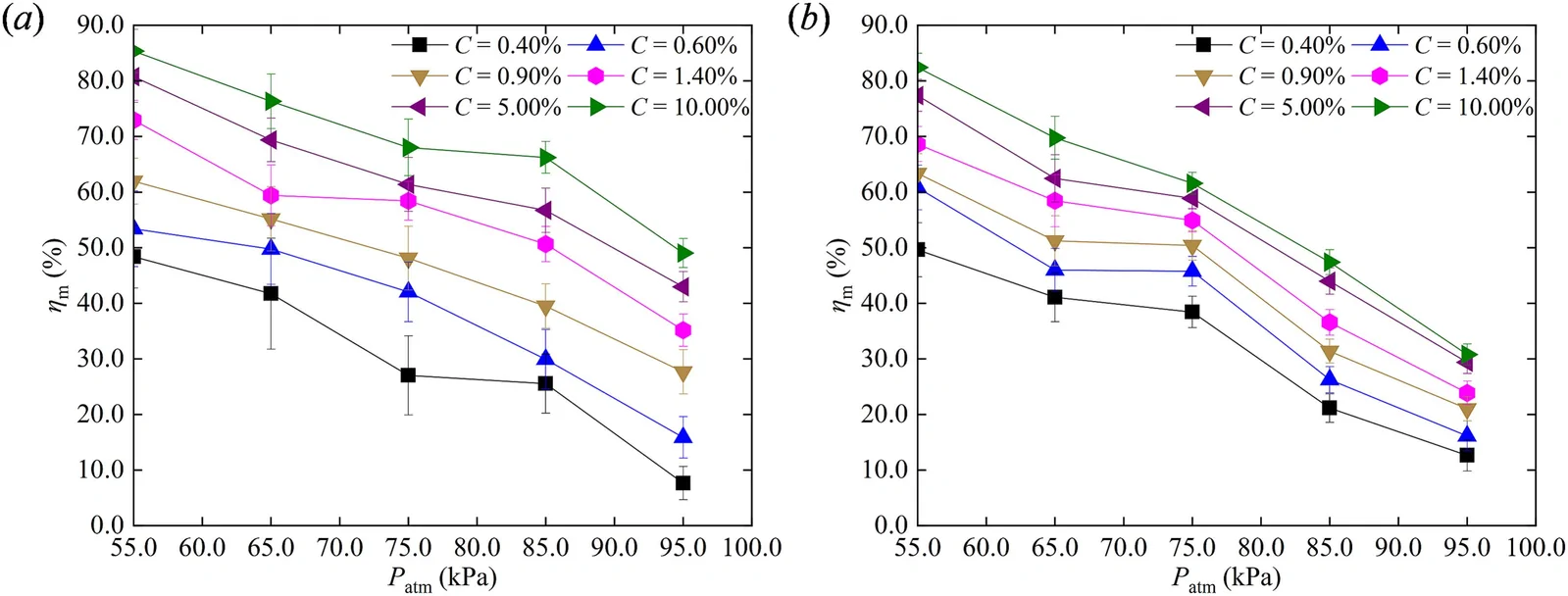
Variation in the cavitation erosion mitigation efficiency of entrained air bubbles with ambient pressure at different cavitation exposure times: (*a*) *T*_e_ = 30 min (*b*) *T*_e_ = 90 min.

The variation in the cavitation erosion mitigation efficiency of entrained air bubbles (*η*_m_) with ambient pressure (*P*_atm_) under different aeration concentrations at two representative cavitation exposure times, *T*_e_ = 30 min and *T*_e_ = 90 min, is shown in Fig. 10.

As shown in Fig. 10, at *T*_e_ = 30 and 90 min, the variation trends of *η*_m_ with *P*_atm_ were generally consistent for different aeration concentrations. At a given *P*_atm_ and *T*_e_, *η*_m_ increased with increasing *C*. Taking *T*_e_ = 90 min and *P*_atm_ = 95 kPa as an example, as *C* increased successively from 0.40% to 0.60%, 0.90%, 1.40%, 5.00%, and 10.00%, *η*_m_ was approximately 12.6%, 16.1%, 21.0%, 23.9%, 29.4%, and 30.8%, respectively. This indicates that, at a given air bubble size, the cavitation erosion mitigation efficiency of entrained air bubbles gradually increased with increasing aeration concentration. However, cavitation erosion was not completely suppressed under the present experimental conditions, even when *C* was increased to 10.00%. In addition, at a given *T*_e_ and *C*, *η*_m_ increased monotonically with decreasing *P*_atm_. For example, at *T*_e_ = 90 min and *C* = 0.40%, as *P*_atm_ was successively reduced from 95 to 85, 75, 65, and 55 kPa, *η*_m_ increased to approximately 21.2%, 38.4%, 41.1%, and 49.6%, respectively.

**Fig. 11:**
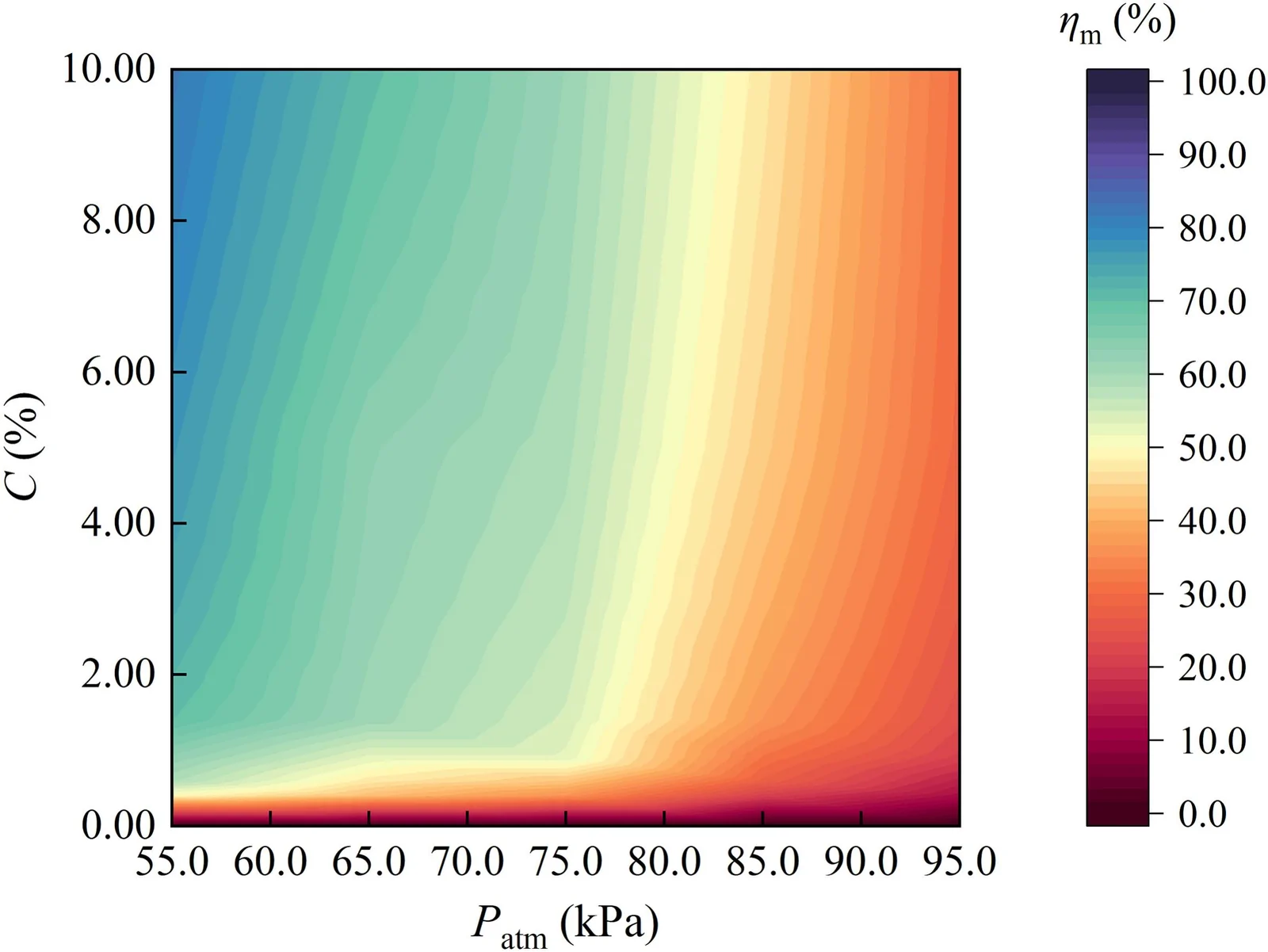
The cavitation erosion mitigation efficiency of entrained air bubbles as a function of aeration concentration and ambient pressure (*T*_e_ = 90 min).

Fig. 11 illustrates how ambient pressure and aeration concentration jointly affect cavitation erosion mitigation. The mitigation efficiency is shown over the full parameter range of *P*_atm_ = 55–95 kPa and *C* = 0%-10.00% at *T*_e_ = 90 min. As can be seen, the mitigation efficiency of entrained air bubbles generally increased with decreasing ambient pressure and increasing aeration concentration. Specifically, at a given *C*, the mitigation efficiency increased monotonically as *P*_atm_ decreased; at a given *P*_atm_, it increased with increasing *C*. Under the conditions of *P*_atm_ = 55 kPa and *C* = 10.00%, the cavitation erosion mitigation efficiency of entrained air bubbles approached 100%.

In summary, based on the quantitative analysis of cavitation erosion pit characteristics, target mass loss, and cavitation erosion rate under different ambient pressures (*P*_atm_ = 55–95 kPa) and aeration concentrations (*C* = 0%-10.00%), two main trends can be identified. First, at a given cavitation exposure time and air bubble size, cavitation erosion damage on the target surface is progressively weakened as aeration concentration increases, when ambient pressure remains unchanged. Second, at the same cavitation exposure time, air bubble size, and aeration concentration, the cavitation erosion mitigation efficiency of entrained air bubbles increases with decreasing ambient pressure, indicating that entrained air bubbles exhibit a more pronounced inhibitory effect on cavitation erosion at lower ambient pressures.

To reveal the intrinsic physical mechanism by which the cavitation erosion mitigation efficiency of aeration gradually increases with decreasing ambient pressure, it is necessary to further investigate how air bubbles affect the collapse dynamics of near-wall cavitation bubbles at the mesoscopic. Previous studies have shown that air bubbles weaken shockwaves generated during cavitation-bubble collapse [28]. Ambient pressure also strongly affects the collapse dynamics of cavitation bubbles, with both the collapse-induced microjet velocity and shockwave intensity decreasing as ambient pressure decreases [16]. However, most existing studies have examined how air bubbles interact with cavitation bubbles under atmospheric pressure conditions. Therefore, to further clarify how entrained air bubbles in gas–liquid two-phase flow affect cavitation bubble collapse behavior under varying ambient pressure conditions, section 5 systematically investigates the related mesoscopic mechanisms. The collapse induced microjets and shockwaves of near-wall cavitation bubbles are analyzed under varying low ambient pressures. The aim is to reveal the mesoscopic mechanism through which reduced ambient pressure changes aeration-based cavitation erosion mitigation efficiency.

## 5 Mesoscopic mechanism analysis

### 5.1 Effect of low ambient pressure on microjets

When a near-wall cavitation bubble collapses, it generates a microjet directed toward the wall, which impinges on the wall at an extremely high velocity and causes significant damage to the material surface [53], [54]. This section examines how entrained air bubbles modify the microjet characteristics during near-wall cavitation bubble collapse and how these characteristics vary with ambient pressure. The aim is to reveal the intrinsic mechanism of cavitation erosion mitigation efficiency of entrained air bubbles under different ambient pressures from the microjet perspective.

Because the sizes of both cavitation bubbles and air bubbles vary with decreasing ambient pressure [16], [55], while the dimensionless form of the Rayleigh-Plesset equation indicates that, under inertia dominated conditions and when other dimensionless parameters remain unchanged or exert only weak effects, bubble dynamics and bubble–bubble interactions exhibit scale similarity [56]. Therefore, their interaction patterns are mainly governed by relative geometric parameters, such as air bubble relative size and cavitation bubble-air bubble separation [22], [23]. To eliminate the interference of air bubble size and highlight the effect of ambient pressure, the experiments were controlled using three dimensionless parameters: *ε*, *δ* and *γ*. Specifically, *ε* denotes the relative air bubble size, *δ* denotes the relative interbubble separation distance, and *γ* denotes the dimensionless bubble-wall standoff distance. Across all ambient pressures, *ε*, *δ* and *γ* were kept constant, with *δ* ≈ 0.80 and *γ* ≈ 0.83. A stand-off parameter of *γ* ≈ 0.83 was selected because the rigid wall exerts a pronounced influence on cavitation bubble evolution at this bubble-wall distance. During collapse, the cavitation bubble forms a well-defined microjet directed toward the wall, facilitating observation of the effects of the air bubble on microjet morphology and velocity. In addition, preliminary experiments showed that, under the present experimental conditions and in the absence of an air bubble, the maximum microjet velocity of near-wall cavitation bubble collapse occurred at *γ* ≈ 0.83, making this condition representative. A bubble-spacing parameter of *δ* ≈ 0.80 was selected to enhance the interaction between the cavitation bubble and the air bubble. This configuration highlights the attenuation of near-wall cavitation bubble collapse by the air bubble and enables its dependence on ambient pressure to be clearly identified.

**Fig. 12:**
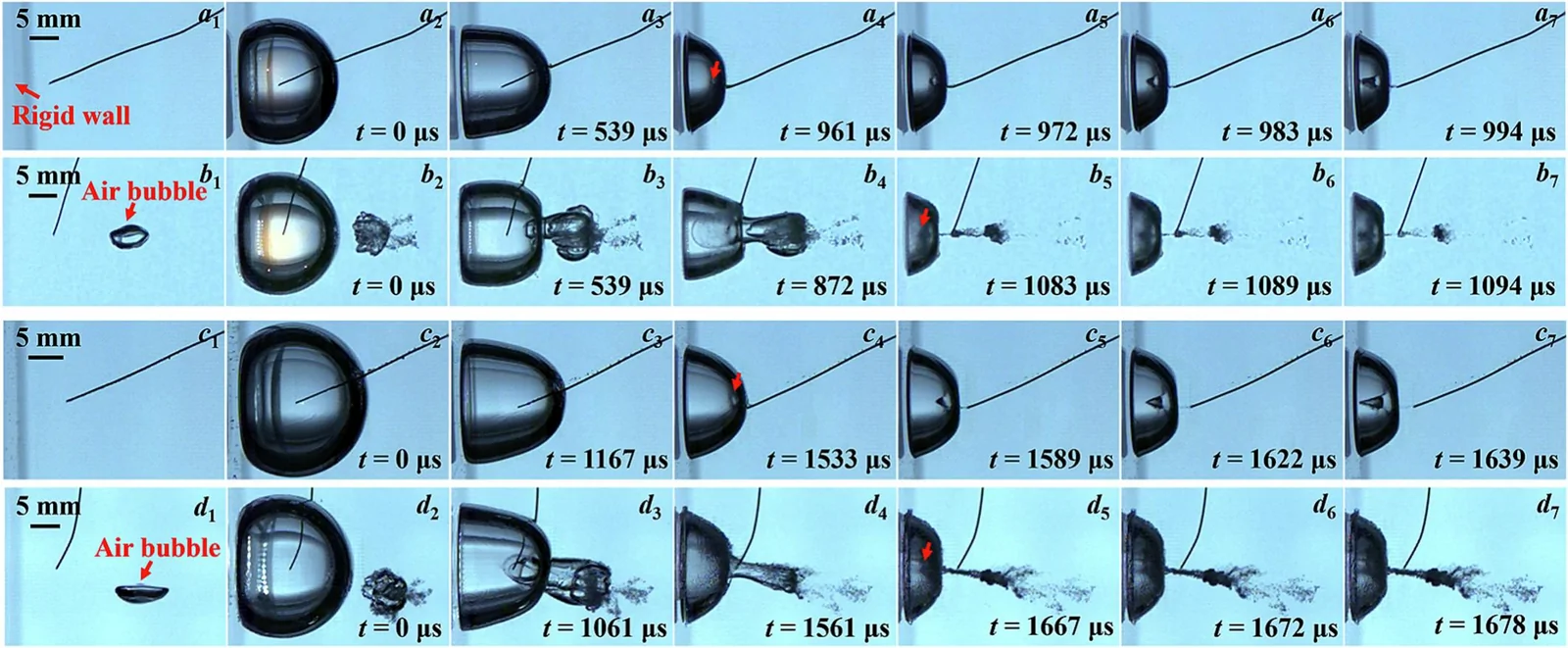
Evolution of microjets from near-wall cavitation bubbles collapse under the influence of air bubbles: (*a*) *P*_atm_ = 95 kPa, *R*_max_ = 9.77 mm, *γ* = 0.83; (*b*) *P*_atm_ = 95 kPa, *R*_max_ = 9.62 mm, *γ* = 0.84, *ε* = 0.29, *δ* = 0.84; (*c*) *P*_atm_ = 55 kPa, *R*_max_ = 11.91 mm, *γ* = 0.82; (*d*) *P*_atm_ = 55 kPa, *R*_max_ = 11.80 mm, *γ* = 0.82, *ε* = 0.30, *δ* = 0.83.

Fig. 12 presents how wall-adjacent cavitation bubbles collapse with and without a nearby air bubble at two representative ambient pressures, *P*_atm_ = 95 kPa and 55 kPa. Under the non-aerated condition at *P*_atm_ = 95 kPa (Fig. 12(a)), the cavitation bubble maintained direct attachment to the wall. During its shrinkage process, the wall-adjacent side of the cavitation bubble remained nearly unchanged, while the opposite side continuously contracted toward the wall. The cavitation bubble shape therefore changed from approximately spherical to progressively flattened, as presented in Fig. 12(*a*_2_)–(*a*_3_). Subsequently, the bubble surface opposite the wall continued to contract, with the microjet tip appearing at *t* = 961 μs (Fig. 12(*a*_4_)). The microjet then developed continuously into a conical shape and impinged toward the wall (Fig. 12(*a*_5_)–(*a*_6_)). At *t* = 994 μs, the jet tip first contacted the wall (Fig. 12(*a*_7_)). Throughout the entire evolution process, the microjet exhibited a distinct morphology with a sharp tip.

With a neighboring air bubble, the collapse behavior of the near-wall cavitation bubble changed markedly (Fig. 12(b)). During cavitation bubble collapse, the air bubble migrated toward the wall and contacted the cavitation bubble surface (Fig. 12(*b*_2_)–(*b*_3_)). It was then drawn into the collapsing cavitation bubble. The microjet formed at *t* = 1083 μs (Fig. 12(*b*_5_)). However, the air bubble had entered the collapsing cavitation bubble, which noticeably disturbed the microjet tip. By *t* = 1094 μs, the microjet struck the wall, and the jet tip showed obvious blunting and fragmentation relative to the non-aerated condition (Fig. 12(*b*_7_)).

Under reduced ambient pressure, cavitation bubbles adjacent to a solid boundary showed collapse behavior generally similar to that at normal pressure condition (*P*_atm_ = 95 kPa), both with and without air bubbles. Fig. 12(c) and (d) present wall-adjacent cavitation bubble collapse without and with an air bubble, respectively, at *P*_atm_ = 55 kPa.

Without an air bubble, the cavitation bubble reached a noticeably larger maximum size at *P*_atm_ = 55 kPa than at *P*_atm_ = 95 kPa, and its collapse duration was significantly prolonged, as presented in Fig. 12(c). This is because reduced ambient pressure lowers the pressure surrounding the cavitation bubble, making its expansion easier and increasing its maximum radius. Meanwhile, during cavitation bubble collapse, the driving pressure difference decreased, which slowed bubble surface contraction and prolonged the collapse duration [16]. At *t* = 1533 μs, a microjet formed and subsequently impinged toward the wall with a distinct conical morphology (Fig. 12(*c*_4_)–(*c*_6_)). At *t* = 1639 μs, the jet contacted the wall, exhibiting a clear morphology and a sharp tip, consistent with the observations at *P*_atm_ = 95 kPa.

With air bubbles introduced, the near-wall cavitation bubble at *P*_atm_ = 55 kPa collapsed in a manner similar to that observed at *P*_atm_ = 95 kPa. During collapse and contraction, the air bubble was drawn into the collapsing cavitation bubble (Fig. 12(*d*_3_)–*d*_4_)). At *t* = 1667 μs, a microjet was observed to form inside the cavitation bubble. The microjet then continued to develop and eventually impacted the wall (Fig. 12(*d*_5_)–(*d*_7_)). Compared with the non-aerated condition, after the air bubble was drawn into the cavitation bubble, the microjet morphology was noticeably disturbed, the conical feature disappeared, and the jet tip became fragmented.

**Fig. 13:**
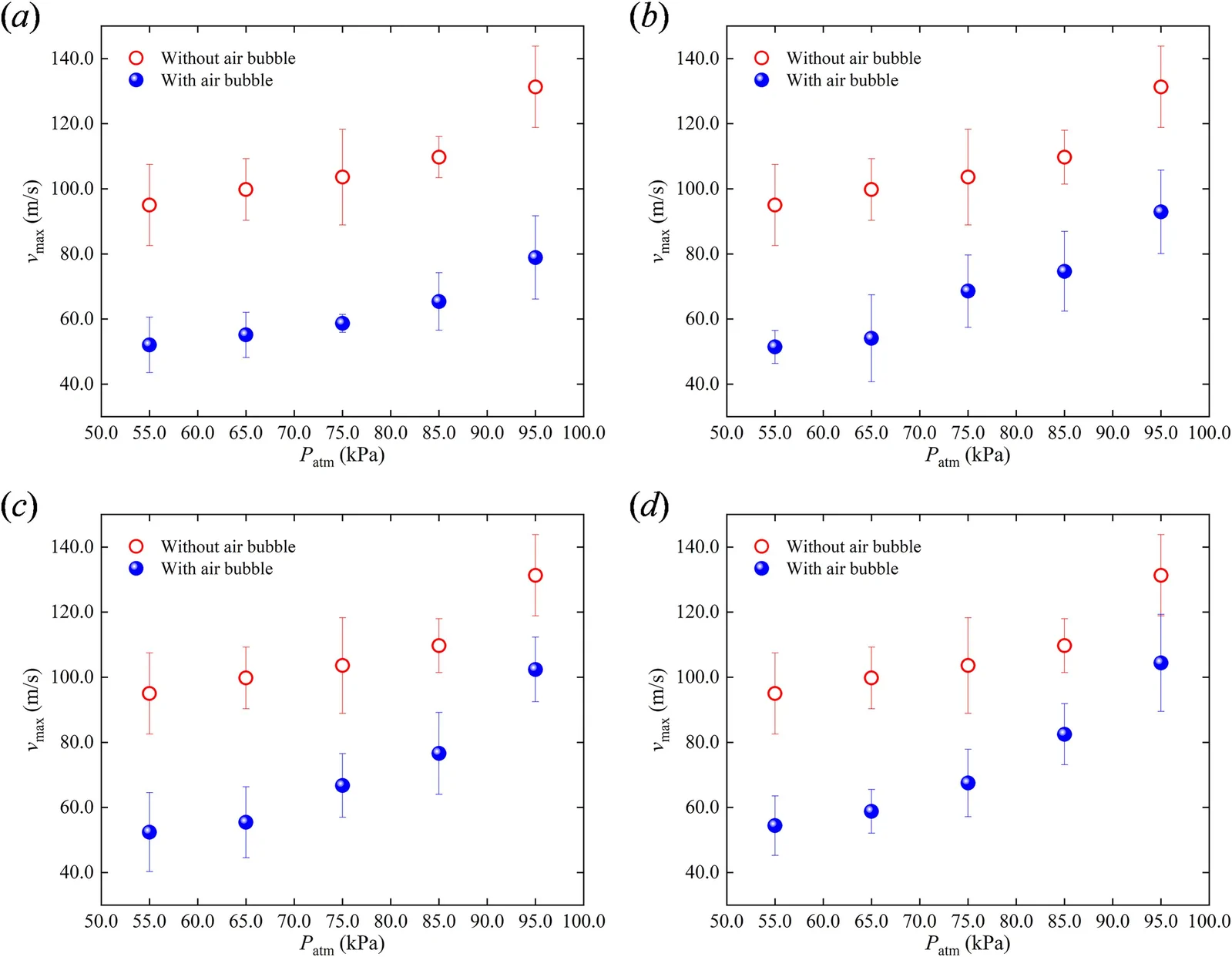
Variation in the maximum microjet velocity with ambient pressure in the absence and presence of an air bubble (*γ* ≈ 0.83, *δ* ≈ 0.80). Panels (*a*)-(*d*) correspond to *ε* ≈ 0.20, 0.30, 0.40, 0.50.

Fig. 13(a)–(d) present the maximum microjet velocity (*v*_max_) as a function of *P*_atm_ at four air bubble relative sizes (*ε* ≈ 0.20, 0.30, 0.40, and 0.50). For comparison, the maximum microjet velocities generated by near-wall cavitation bubble collapse at *γ* ≈ 0.83 without an air bubble under different ambient pressures were added to all four panels, as indicated by the red points. It can be shown that *v*_max_ increased with *P*_atm_ in the non-aerated condition. This indicates that ambient pressure strongly affects near-wall cavitation bubble collapse dynamics and that lower ambient pressure reduces the maximum microjet speed. At a given *P*_atm_, the air bubble significantly reduced *v*_max_. For example, at *P*_atm_ = 95 kPa and *ε* ≈ 0.30, the mean value of *v*_max_ was approximately 131.3 m/s in the absence of an air bubble but decreased to approximately 92.9 m/s after the introduction of an air bubble, corresponding to a reduction of approximately 29.2%, as presented in Fig. 13(b).

Moreover, the ability of air bubbles to reduce the maximum microjet velocity varied markedly with ambient pressure during wall-adjacent cavitation-bubble collapse. This variation is mainly reflected in two aspects. First, in the presence of an entrained air bubble, the maximum microjet velocity gradually decreased with decreasing ambient pressure. Taking *ε* ≈ 0.20 as an example, the value of *v*_max_ in the presence of an air bubble was 78.9 m/s at *P*_atm_ = 95 kPa to 52.0 m/s at *P*_atm_ = 55 kPa, corresponding to a decrease of approximately 34.1%. Second, air bubbles became more effective in slowing microjets as ambient pressure decreased. For example, at *ε* ≈ 0.30, the reduction efficiency of air bubbles for the mean value of *v*_max_, denoted as *η*_vm_, was approximately 29.2% at *P*_atm_ = 95 kPa, whereas it increased to approximately 45.6% at *P*_atm_ = 55 kPa. Here, *η*_vm_ is defined as *η*_vm_ = (*v*_max,0_-*v*_max,air_)/*v*_max,0_ × 100%, where *v*_max,0_ and *v*_max,air_ are the mean maximum velocities of microjet induced by the collapse of cavitation bubbles adjacent to a wall without and with an air bubble, respectively. These results suggest that, under the same aeration condition, the cavitation erosion mitigation efficiency of entrained air bubbles gradually increases with decreasing ambient pressure.

The above trends can be explained from the following two aspects. First, when *P*_atm_ and *ε* are kept constant and *δ* is relatively small, the cavitation bubble entrains the air bubble into its interior during collapse. The increased non-condensable gas content raises the internal gas pressure and reduces the effective driving pressure difference. As a result, cavitation bubble contraction is weakened, and the microjet velocity decreases. In addition, the entrained air bubble forms a deformable cushion with low acoustic impedance inside the cavitation bubble. This disturbs microjet development and changes its morphology, leading to blunting and fragmentation of the microjet tip. Consequently, the maximum microjet velocity is further reduced. Second, without air bubbles, decreasing ambient pressure reduces the pressure surrounding the cavitation bubble. This weakens the driving pressure difference and thus lowers the microjet velocity. When an air bubble is present, low ambient pressure promotes its expansion and strengthens the compressible cushioning effect. After the two bubbles coalesce, the contraction of the wall-adjacent cavitation bubble is further suppressed, and the microjet velocity decreases. As a result, the reduction efficiency of air bubbles for the maximum microjet velocity continuously increases as ambient pressure decreases.

### 5.2 Effect of low ambient pressure on shockwaves

Shockwaves are a major cause of cavitation erosion during near-wall cavitation bubble collapse [57], [58]. This section examines how entrained air bubbles affect the shockwave characteristics generated as cavitation bubbles collapse close to a wall and how these characteristics vary with ambient pressure. To eliminate interference from extraneous variables while ensuring measurement repeatability, *γ* and *δ* were held constant across all ambient pressures (*γ* ≈ 2.00 and *δ* ≈ 0.80). The selection of *γ* ≈ 2.00 was based mainly on two considerations. First, at this bubble-wall distance, the microjet is unlikely to directly impact the wall, allowing the damaging effect of the collapse-induced shockwave on the wall to be more clearly identified. Second, to ensure the repeatability of the pressure measurements, a sufficient distance was maintained between the cavitation bubble and the wall-mounted pressure sensor. This prevented the cavitation bubble from directly contacting and potentially damaging the sensor surface, which could otherwise compromise the accuracy of the measurements.

**Fig. 14:**
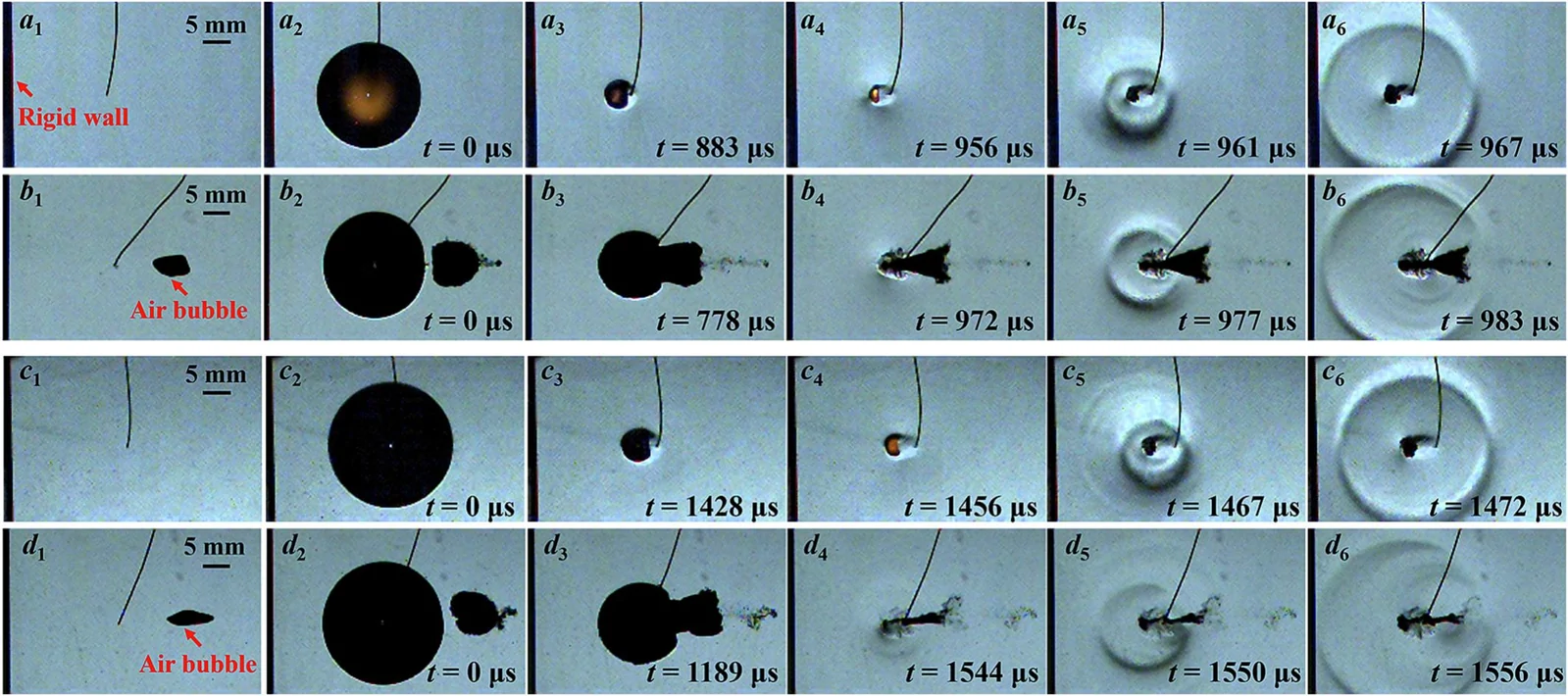
Evolution of collapse-induced shockwaves from wall-adjacent cavitation bubbles affected by air bubbles: (*a*) *P*_atm_ = 95 kPa, *R*_max_ = 9.90 mm, *γ* = 2.02; (*b*) *P*_atm_ = 95 kPa, *R*_max_ = 9.63 mm, *γ* = 2.04, *ε* = 0.30, *δ* = 0.76; (*c*) *P*_atm_ = 55 kPa, *R*_max_ = 11.82 mm, *γ* = 2.03; (*d*) *P*_atm_ = 55 kPa, *R*_max_ = 11.71 mm, *γ* = 2.02, *ε* = 0.26, *δ* = 0.81.

Fig. 14 presents the evolution of shockwaves generated during near-wall cavitation bubble collapse with nearby air bubbles at different ambient pressures. Fig. 14(a) shows how shockwaves evolved when a wall-adjacent cavitation bubble collapsed at *P*_atm_ = 95 kPa. As can be observed, the cavitation bubble contracted to its first-cycle minimum radius at *t* = 956 μs, accompanied by distinct luminescence (Fig. 14(*a*_4_)). This luminescence results from strong gas compression within the collapsing bubble, which drives a localized concentration of energy [59]. Subsequently, shockwaves propagated radially outward in all directions (Fig. 14(*a*_5_)-(*a*_6_)). When air bubble was present in the vicinity of the near-wall cavitation bubble, the collapse process differed significantly from that under the non-aerated condition (Fig. 14(b)). During collapse, the air bubble was drawn into the cavitation bubble (Fig. 14(*b*_3_)). At the first-cycle minimum radius (*t* = 972 μs), no obvious luminescence was observed (Fig. 14(*b*_4_)), suggesting a marked reduction in the collapse intensity of the cavitation bubble due to the air bubble. Shockwaves then propagated outward at *t* = 977 μs.

After ambient pressure was reduced, the wall-adjacent cavitation bubbles collapse behavior differed from that observed under normal pressure conditions, both with and without an air bubble. The collapse process of a wall-adjacent cavitation bubble in the absence of an air bubble at *P*_atm_ = 55 kPa is shown in Fig. 14(c). The cavitation bubble contracted to its smallest first-cycle volume at *t* = 1456 μs. At this moment, the luminescence intensity was noticeably weaker than that at *P*_atm_ = 95 kPa, indicating that the intrinsic collapse intensity was reduced after the decrease in ambient pressure. With an air bubble, the collapse and contraction process of the wall-adjacent cavitation bubble at *P*_atm_ = 55 kPa was similar to that observed under the corresponding condition at *P*_atm_ = 95 kPa (Fig. 14(d)). During collapse stage, the air bubble was drawn into the cavitation bubble. No obvious luminescence appeared when the cavitation bubble reached its minimum volume.

**Fig. 15:**
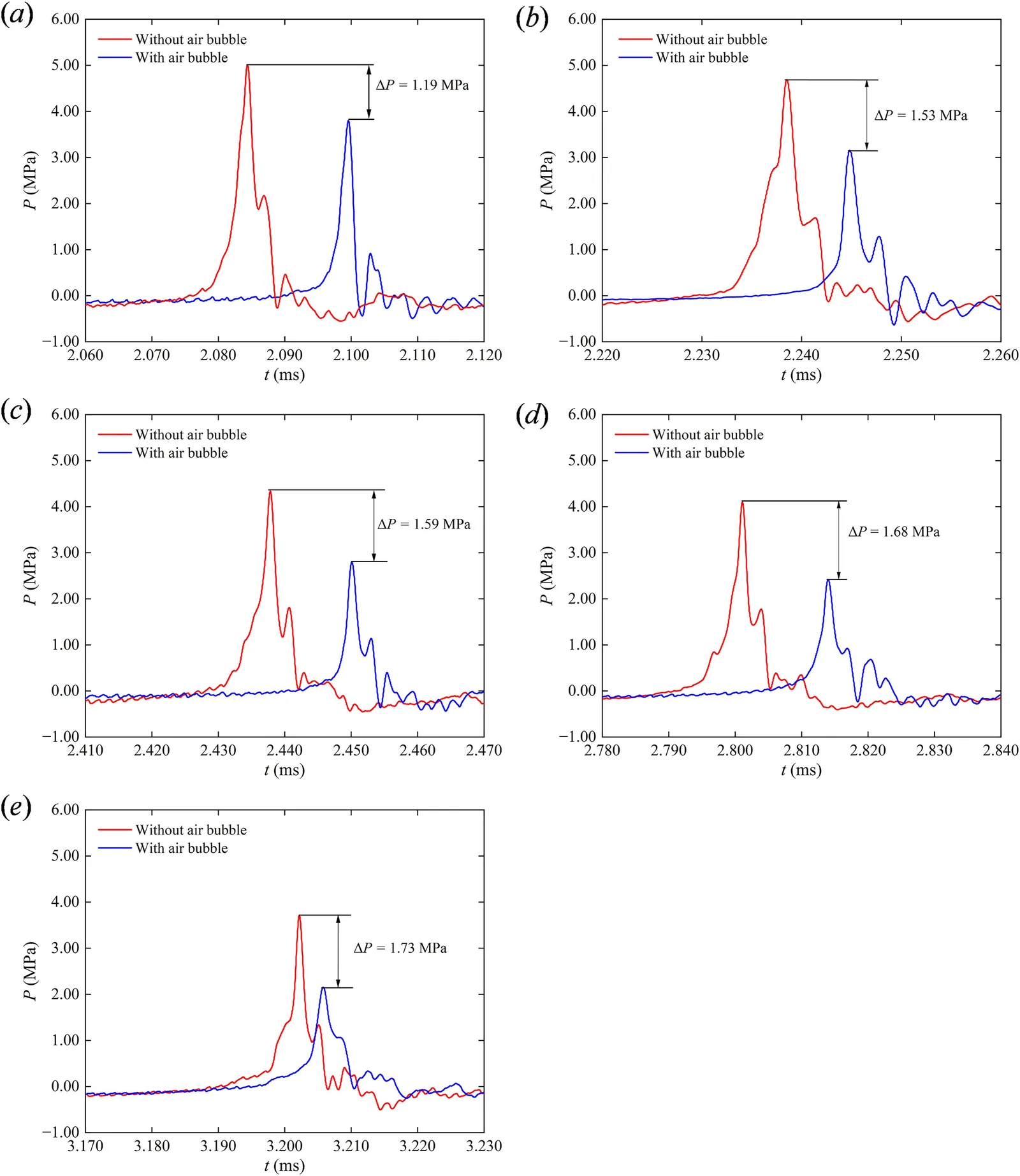
Shockwave pressure signals generated by the first collapse of a near-wall cavitation bubble in the presence of an air bubble (*γ* ≈ 2.00, *δ* ≈ 0.80, *ε*≈ 0.30). Panels (*a*)-(*e*) correspond to *P*_atm_ = 95, 85, 75, 65, and 55 kPa, respectively.

The shockwave pressure of the near-wall cavitation bubble first collapse was further measured quantitatively using a high-frequency pressure sensor at different ambient pressures, both with and without an air bubble. The resulting pressure–time histories are shown in Fig. 15. For ease of comparison between the peaks pressure obtained with and without an air bubble, the two curves in each panel were shifted horizontally relative to each other. Therefore, the time coordinates on the horizontal axis have no physical significance. Comparison of the pressure–time histories at different ambient pressures (Fig. 15(a)–(e)) reveals two main trends. First, in the absence of an air bubble and at the same dimensionless cavitation bubble-rigid wall distance, the peak pressure of shockwave from wall-adjacent cavitation bubbles collapse decreased as the ambient pressure decreased. Second, at each ambient pressure, the peak pressure was substantially lower in the presence of an air bubble than in its absence. Moreover, the relative reduction increased as the ambient pressure decreased. These results indicate that, for comparable air bubble characteristics, the efficiency of the air bubble in reducing the shockwave peak pressure increased with decreasing ambient pressure.

The peak pressure (*P*_max_) exerted on the wall by the shockwaves from wall-adjacent cavitation bubble collapse was further quantitatively recorded under conditions with and without an air bubble.

**Fig. 16:**
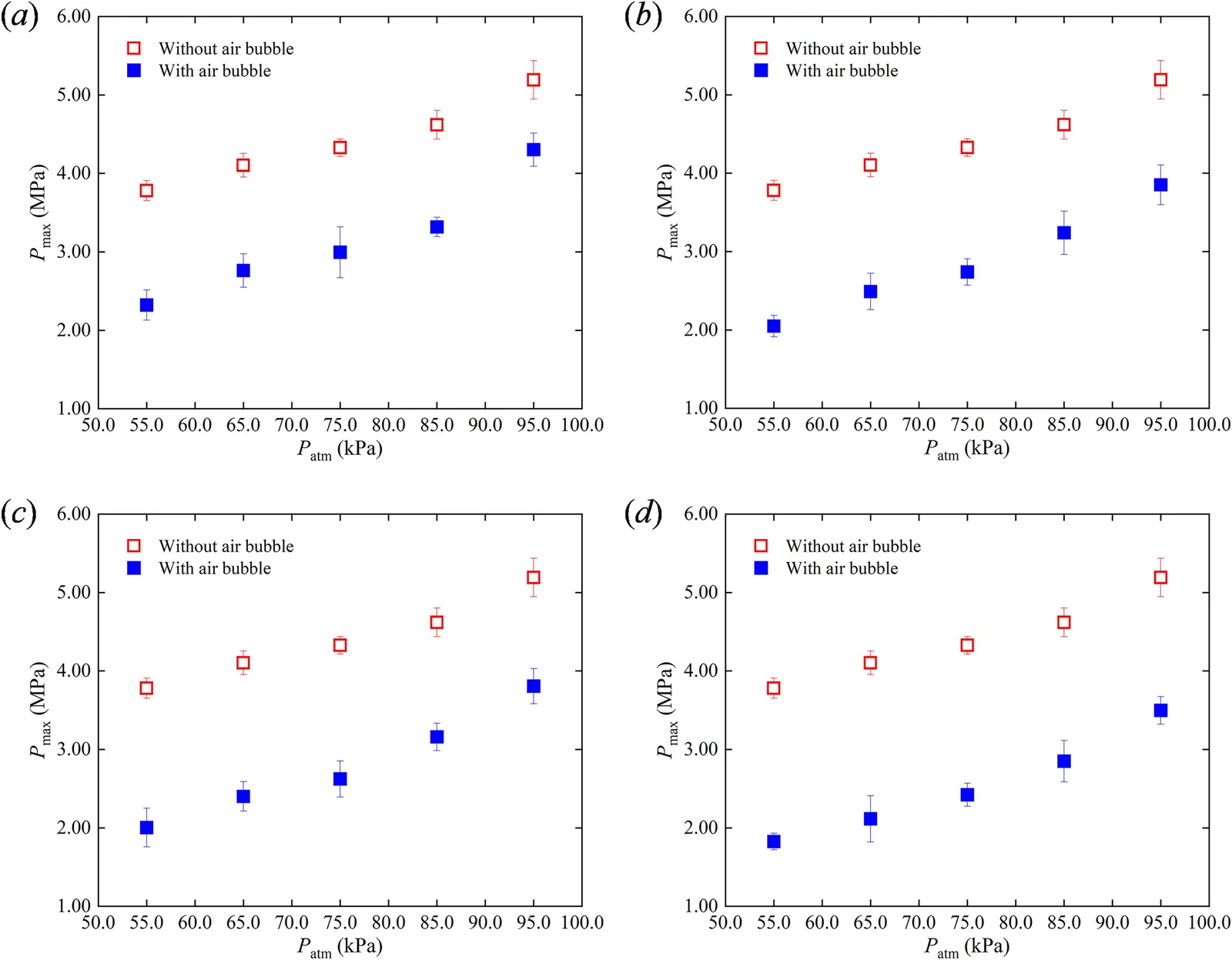
Effect of ambient pressure on wall-impinging shockwave peak pressure during wall-adjacent cavitation bubble collapse under aerated and non-aerated conditions (*γ* ≈ 2.00, *δ* ≈ 0.80). Panels (*a*)-(*d*) correspond to *ε* ≈ 0.20, 0.30, 0.40, 0.50.

In this study, *P*_max_ is defined as the shockwave peak pressure during the first collapse of the cavitation bubble. It was determined as the maximum positive pressure recorded by the pressure sensor among the distinct pressure pulses associated with the cavitation bubble first collapse. Fig. 16 presents how *P*_max_ changes with ambient pressure (*P*_atm_). For comparison, the peak pressure of the shockwave generated by the collapse of a near-wall cavitation bubble at *γ* ≈ 2.00 without an air bubble were included in all four panels for the different ambient pressures, as indicated by the red symbols.

As can be observed, without an air bubble, *P*_max_ decreased with decreasing *P*_atm_, indicating that the shockwave intensity weakened as ambient pressure decreased. At a given *P*_atm_, an air bubble significantly reduced *P*_max_ compared with the non-aerated condition. For example, at *P*_atm_ = 95 kPa and *ε* ≈ 0.30, *P*_max_ decreased from 5.19 MPa without an air bubble to a mean value of 3.87 MPa with an air bubble, corresponding to a reduction of approximately 25.4%. This indicates that air bubbles can significantly attenuate the intensity of shockwaves generated by near-wall cavitation bubble collapse.

Furthermore, ambient pressure significantly affected how air bubbles attenuated the shockwave peak pressure, mainly in two aspects. First, in the presence of an air bubble, the shockwave peak pressure gradually decreased with decreasing ambient pressure. Taking *ε* ≈ 0.30 as an example, the mean value of *P*_max_ was 3.87 MPa at *P*_atm_ = 95 kPa, whereas it decreased to 2.15 MPa when *P*_atm_ was reduced to 55 kPa, showing an approximately 44.4% decrease compared with that at *P*_atm_ = 95 kPa. Second, the reduction efficiency of air bubbles on *P*_max_ gradually increased as ambient pressure decreased. For example, at *ε* ≈ 0.30, the reduction efficiency of air bubbles on *P*_max_, denoted as *η*_pm_, was approximately 25.4% at *P*_atm_ = 95 kPa, whereas it increased to approximately 43.1% at *P*_atm_ = 55 kPa. Here, *η*_pm_ is defined as *η*_pm_ = (*P*_max0_–*P*_max,air_)/*P*_max0_ × 100%, where *P*_max0_ and *P*_max,air_ denote the mean shockwave peak pressure measured without and with an air bubble, respectively. These results demonstrate that, under otherwise identical aeration characteristics, the cavitation erosion mitigation efficiency of entrained air bubbles gradually increases with decreasing ambient pressure.

The air bubble lowers the shockwave peak pressure. The corresponding reduction efficiency also increases as ambient pressure decreases. These trends can be explained from the following two perspectives. First, when *δ* is relatively small, the cavitation bubble coalesces with the air bubble during collapse. The non-condensable gas introduced by the air bubble raises the cavitation bubble internal pressure during collapse and reduces the effective driving pressure difference. As a result, the contraction velocity of the cavitation bubble surface decreases, the collapse intensity is weakened, and the intensity of the corresponding shockwaves is reduced. Second, as ambient pressure decreases, internal-external pressure difference driving cavitation bubble collapse is reduced, leading to weaker intrinsic collapse intensity. After the two bubble coalesces and incorporates non-condensable gas, the internal-external driving pressure difference of the cavitation bubble becomes lower than that under atmospheric pressure conditions. This further weakens the collapse intensity of the cavitation bubble, thereby causing the reduction efficiency of air bubbles for shockwaves intensity to gradually increase with decreasing ambient pressure.

Based on the mesoscopic experimental results presented in 5.1, 5.2, the mechanism underlying the macroscopic increase in the cavitation erosion mitigation efficiency of entrained air bubbles with decreasing ambient pressure can be summarized from the following three perspectives.

First, compared with the non-aerated condition, air bubbles significantly affect the collapse-induced microjet and shockwave characteristics of near-wall cavitation bubbles. Specifically, the maximum microjet velocity decreases, and the shockwave peak pressure impinging on the wall is attenuated. As ambient pressure decreases, both quantities are further reduced in the presence of air bubbles, and the corresponding reduction efficiencies increase progressively with decreasing ambient pressure. Second, cavitation bubble collapse intensity changes markedly with ambient pressure. With a reduction in ambient pressure, both the collapse-induced microjet velocity of near-wall cavitation bubbles and the shockwave peak pressure on the wall decrease [16], [60]. Third, at lower ambient pressure, entrained air bubbles become larger than those under atmospheric pressure, and their volume oscillation amplitude increases [55]. This enhances their influence on wall-adjacent cavitation-bubble collapse and further reduces the collapse intensity under low ambient pressure conditions. Moreover, from a practical engineering perspective, when entrained air bubble population remains constant, the increase in bubble volume caused by reduced ambient pressure is, to some extent, equivalent to an increase in the aeration concentration of the flow.

Collectively, these three aspects reveal the intrinsic microscale mechanism by which the efficiency of aeration-based cavitation erosion mitigation gradually increases as ambient pressure decreases in practical engineering applications.

## 6 Conclusion

This study focuses on the effect of low ambient pressure (below 1.013 × 10^5^ Pa) on the efficiency of aeration in mitigating cavitation erosion. Over an ambient pressure range of 55–95 kPa, ultrasonic cavitation experiments, corona discharge-induced cavitation bubble system, ultra-high-speed camera and high-frequency pressure sensor were employed to systematically investigate the effects of ambient pressure and air concentration on cavitation erosion mitigation efficiency while keeping the air bubble size approximately constant. At the mesoscale, the effects of an air bubble on the microjet and shockwave characteristics of near-wall cavitation bubble collapse, as well as their variation with ambient pressure, were also elucidated.

The macroscale experimental results showed that the efficiency of aeration in mitigating cavitation erosion increased as the ambient pressure decreased. With the air bubble size, air concentration, and cavitation conditions kept nearly identical, the eroded-area ratio, pit depth, mean pit volume, cumulative mass loss, and cavitation-induced mass loss rate all decreased with decreasing ambient pressure. Moreover, the reduction efficiencies of entrained air bubbles for these erosion parameters gradually increased progressively as the ambient pressure decreased. At a given ambient pressure, increasing the air concentration further reduced all erosion parameters and enhanced the efficiency of aeration in mitigating cavitation erosion.

The mesoscale experimental results showed that, in the presence of an air bubble, both the maximum microjet velocity generated by near-wall cavitation bubble collapse and the shockwave peak pressure decreased markedly as the ambient pressure decreased. When the air bubble characteristics were kept approximately consistent, the reduction efficiencies of air bubbles for both the maximum microjet velocity and the shockwave peak pressure gradually increased with decreasing ambient pressure. These findings provide a preliminary mechanistic explanation for the macroscale observation that the efficiency of aeration in mitigating cavitation erosion increases as the ambient pressure decreases.

The present findings provide experimental evidence for understanding the influence of low ambient pressure on the efficiency of aeration in mitigating cavitation erosion and the associated mesoscale mechanisms. They may also provide a theoretical reference for the application of aeration-based cavitation erosion mitigation in high-altitude, low atmospheric pressure environments. However, the experiments on the interaction between a single cavitation bubble and an air bubble were conducted under controlled bubble-wall distances, interbubble distances and relative bubble sizes, and therefore do not encompass the full complexity of actual aerated cavitation erosion fields. The mechanisms identified in this study require further validation under conditions more representative of prototype applications.

## CRediT authorship contribution statement

**Tong Qu:** Writing – original draft, Visualization, Methodology, Investigation, Formal analysis. **Jing Luo:** Writing – original draft, Methodology, Formal analysis. **Jie Li:** Investigation, Formal analysis. **Guihua Fu:** Investigation. **Huamei Yang:** Investigation. **Weilin Xu:** Supervision, Methodology, Conceptualization.

## Declaration of competing interest

The authors declare that they have no known competing financial interests or personal relationships that could have appeared to influence the work reported in this paper.

## Appendix A Supplementary data

Supplementary Data 1 High-resolution versions of the figures presented in the article.
